# The Memory of Rice Response to Spaceflight Stress: From the Perspective of Metabolomics and Proteomics

**DOI:** 10.3390/ijms23063390

**Published:** 2022-03-21

**Authors:** Deyong Zeng, Jie Cui, Yishu Yin, Yi Xiong, Wenchen Yu, Haitian Zhao, Shuanghong Guan, Dayou Cheng, Yeqing Sun, Weihong Lu

**Affiliations:** 1 Department of Food Science and Engineering, School of Chemistry and Chemical Engineering, Harbin Institute of Technology, Harbin 150001, China; 18B925086@stu.hit.edu.cn (D.Z.); cuijie2006@163.com (J.C.); 19B925086@stu.hit.edu.cn (Y.Y.); 17B925075@stu.hit.edu.cn (Y.X.); 21B925065@stu.hit.edu.cn (W.Y.); zhaoht9999@163.com (H.Z.); workhard@hit.edu.cn (S.G.); hgdcdy@126.com (D.C.); 2 National and Local Joint Engineering Laboratory for Synthesis, Transformation and Separation of Extreme Environmental Nutrients, Harbin Institute of Technology, Harbin 150001, China; 3 The Intelligent Equipment Research Center for the Exploitation of Characteristic Food & Medicine Resources, Chongqing Research Institute, Harbin Institute of Technology, Chongqing 401135, China; 4 Institute of Environmental Systems Biology, School of environmental science and engineering, Dalian Maritime University, Dalian 116026, China; yqsun@dlmu.edu.cn

**Keywords:** rice, iTRAQ, metabolomics, spaceflight

## Abstract

The stress response of plants to spaceflight has been confirmed in contemporary plants, and plants retained the memory of spaceflight through methylation reaction. However, how the progeny plants adapt to this cross-generational stress memory was rarely reported. Here, we used the ShiJian-10 retractable satellite carrying Dongnong416 rice seeds for a 12.5-day on-orbit flight and planted the F2 generation after returning to the ground. We evaluated the agronomic traits of the F2 generation plants and found that the F2 generation plants had no significant differences in plant height and number of tillers. Next, the redox state in F2 plants was evaluated, and it was found that the spaceflight broke the redox state of the F2 generation rice. In order to further illustrate the stress response caused by this redox state imbalance, we conducted proteomics and metabolomics analysis. Proteomics results showed that the redox process in F2 rice interacts with signal transduction, stress response, and other pathways, causing genome instability in the plant, leading to transcription, post-transcriptional modification, protein synthesis, protein modification, and degradation processes were suppressed. The metabolomics results showed that the metabolism of the F2 generation plants was reshaped. These metabolic pathways mainly included amino acid metabolism, sugar metabolism, cofactor and vitamin metabolism, purine metabolism, phenylpropane biosynthesis, and flavonoid metabolism. These metabolic pathways constituted a new metabolic network. This study confirmed that spaceflight affected the metabolic changes in offspring rice, which would help better understand the adaptation mechanism of plants to the space environment.

## 1. Introduction

In order to further explore deep space and take advantage of the resources of other parts of the solar system, many countries have proposed space missions such as manned landing on the moon, landing on Mars or even the construction of planetary bases [1]. Unlike the terrestrial environment, the effects of the space environment on plants are mainly microgravity, space radiation, magnetic fields, and the final combined effects of these factors on gas exchange, photosynthesis, and water and solute transport [2]. With the growing interest in space agriculture, scientists have been working to better understand the effects of the space environment on various plants in the past five years. In the highly closed system of the space environment, a better understanding of hydroponic nutrient solutions makes it possible to better grow plants in space [3]. In addition, food self-sufficiency was necessary to support astronauts during long-term space missions, and plant biology has made it possible to provide astronauts with fresh food [4].

Current research has confirmed that spaceflight would produce a stress response to plants. After a long period of spaceflight, rice seeds showed no germination or were undeveloped or elongated after germination without cell division [5]. The somatic cells of corn seeds mutated after spaceflight, which led to white and yellow stripes, leaf color changes, and dwarfing in the plant [6]. After wheat seeds were planted on the International Space Station, wheat grains showed significant changes in grain weight. In addition, the spatial environment also affected the bristle cells, tube cells, and transverse cells of wheat [7]. In the past few decades, our team has sent 50 rice lines seeds using Chinese recyclable satellites and spacecraft. After the spacecraft landed, rice seeds were planted in laboratories and fields, then phenotypic variation, cytological effects, and genome mutation characteristics, as well as further changes in phenotype, cytology, molecular level, protein profile, and genome methylation, were all analyzed [8]. Under abiotic stress on the ground, the level of chromatin condensation and decondensation and the shape of other nuclear domains are affected [9], which will affect the expression modification of genes, and ultimately lead to the affected expression abundance of proteins. Moreover, changes in magnetic fields can affect nucleolar and chromosome structure, levels of chromatin condensation and decondensation, resulting in delayed ribosome processing [10]. Therefore, the changes in rice epigenetics and protein synthesis rate after spaceflight may be related to the changes in the magnetic field and the chromatin condensation process during spaceflight. Our previous research results have shown that spaceflight has an impact on contemporary rice’s energy metabolism, amino acid metabolism, sugar metabolism, signal transduction processes, etc., and confirmed that this effect was triggered by ROS [11,12,13]. The differences in epigenetic regulation of gene expression induced by environmental stress exposure can continue to the next generation of stressed plants [14]. In some cases, these intergenerational influences can even be passed on to at least two generations of seeds [15]. Recently, DNA methylation has been proved to be the potential evidence of spaceflight in Arabidopsis intergenerational inheritance [16], and it was believed that DNA methylation induced the expression of a variety of abiotic stress genes in Arabidopsis progeny, including stress response, cold response, cell wall remodeling, nitrate metabolism, plant hormones, Ca^2+^ signal transduction, etc. [16]. The previous work of our team also confirmed that spaceflight caused the methylation of the rice genome, and the changes in methylation were more obvious in the representative variants [8]. Therefore, we speculated that spaceflight can also be inherited across generations in rice. Then, how the progeny rice adapted to the spaceflight by regulating its own metabolism and whether it maintained a consistent mechanism during the different growth and development stages of the progeny need to be reported urgently.

To clarify, the intergenerational inheritance of spaceflight in rice will help to better reveal the intrinsic mechanism of spaceflight mutagenesis. Here, we reported how the offspring rice responded to spaceflight, and even if there was no direct processing of spaceflight in the F2 generation, some changes were still inherited. This research provides a new perspective for a better understanding of the genetic effects of spaceflight.

## 2. Result

### 2.1. The Effect of Spaceflight on the Morphology and Physiology of Offspring Rice

**Figure 1:**
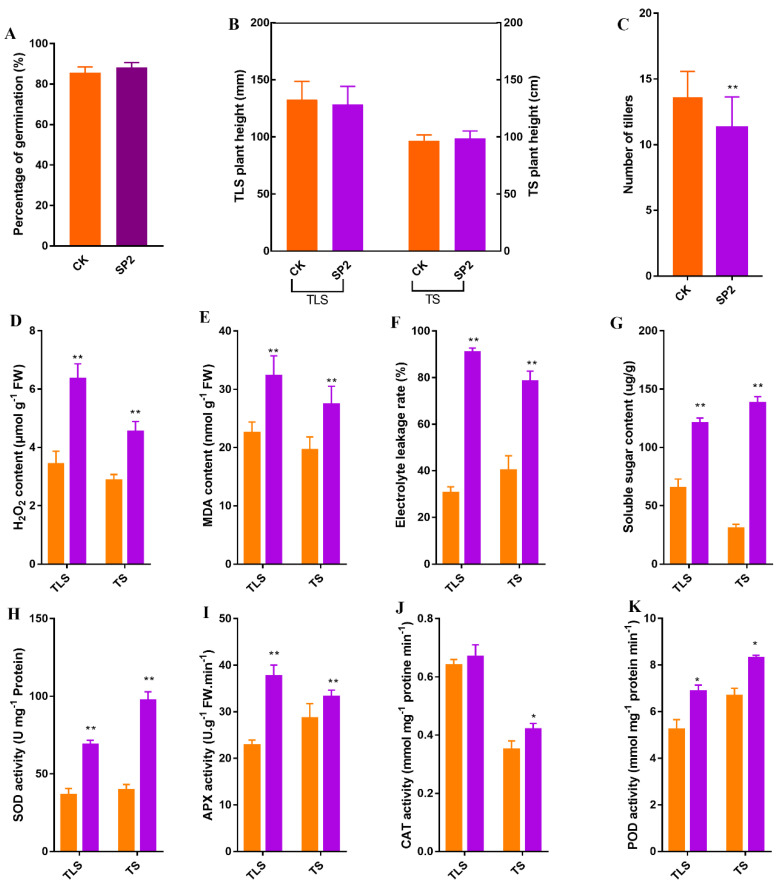
The morphology and physiology of offspring rice induced by spaceflight. (**A**) Percentage of germination. (**B**) Plant height. (**C**) Number of tillers. TLS, three-leaf stage; TS, tillering stage; the orange column represents the control group; the purple column represents the spaceflight group. (**D**) H_2_O_2_ content. (**E**) MDA content. (**F**) The electrolyte leakage rate. (**G**) The soluble sugar contents. (**H**) SOD activity. (**I**) APX activity. (**J**) CAT activity. (**K**) POD activity. Data are expressed as mean ± SD, *n* = 30 (**A**–**C**). The data (mean ± SD) are the means of three replicates with standard errors shown by vertical bars, *n* = 3 (**D**–**K**), * and ** indicate significant difference at *p* < 0.05 and <0.01 by Tukey’s test, respectively.

First, we evaluated the changes in some traits of offspring rice. Compared with the control group, the germination rate of the F2 generation spaceflight group (SP2) did not change significantly (Figure 1A). There was no significant change in plant height during three-leaf stage (TLS) and tillering stage (TS) (Figure 1B). However, at TS, the number of tillers in the SP2 group was significantly reduced (Figure 1C).

In order to preliminarily evaluate whether the spaceflight caused the stress response of offspring rice, we determined the physiological indicators used to characterize the stress response. In this study, H2O2, MDA, electrolyte leakage rate (EL), and soluble sugar (SSC) contents increased significantly in the SP2 group of TLS and TS (Figure 1D–G). In addition, our results also showed that the antioxidant enzyme (superoxide dismutase, ascorbate peroxidase, peroxidase) activity in the SP2 group increased at two different growth and development stages (Figure 1H,I,K). The activity of catalase (CAT) in the SP2 group only increased significantly in the TS stage, and there was no significant change in the TLS stage (Figure 1J).

### 2.2. Changes in Offspring Rice Proteome Profile under Spaceflight

**Figure 2:**
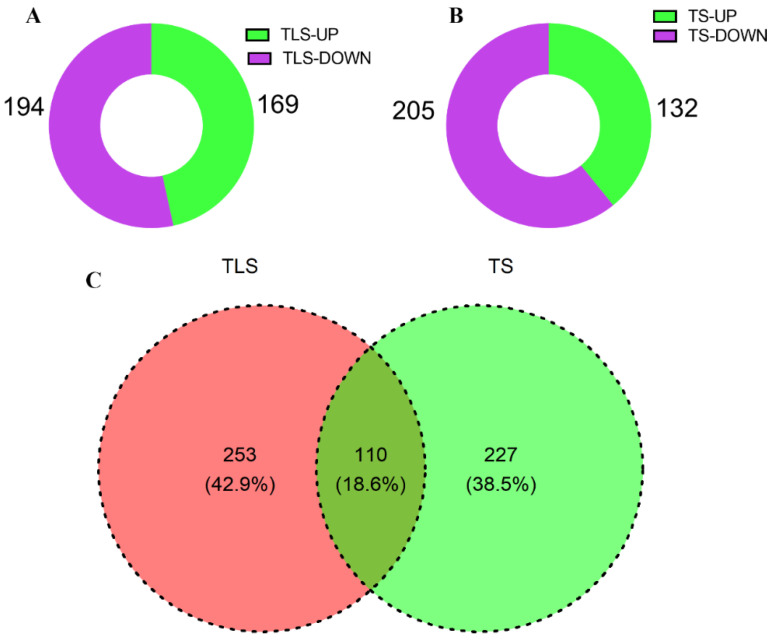
Statistical analysis of DAPS of spaceflight in different growth stages of rice progeny. (**A**) represents the DAPs at the TLS; (**B**) represents the DAPs at the TS; (**C**) represents the Venn diagram of the DAPs at the TLS and the TS. TLS and TS stand for three-leaf stage and tillering stage, respectively.

To study the response program of offspring rice to the spaceflight, we used iTRAQ proteomics marker technology to quantitatively study the protein changes in offspring rice at different growth and development stages. In this study, a total of 3674 proteins were identified. We exceeded 1.2-fold-change and low 1/1.2-fold-change as the up- and downregulated standards. In the TLS and the TS, there were 363 and 337 differentially abundant proteins (DAPs) (Figure 2, Table S1), respectively. Among them, 169 DAPs were upregulated and 194 DAPs were downregulated at the TLS (Figure 2A). At the TS, 132 DAPs were upregulated and 205 DAPs were downregulated (Figure 2B, Table S2). There were 253 and 227 unique DAPs in the TLS and the TS, respectively, and 110 DAPs were shared in two different growth and development stages (Figure 2C).

### 2.3. Protein Level Insight into the Effect of Spaceflight on Rice Progeny

**Figure 3:**
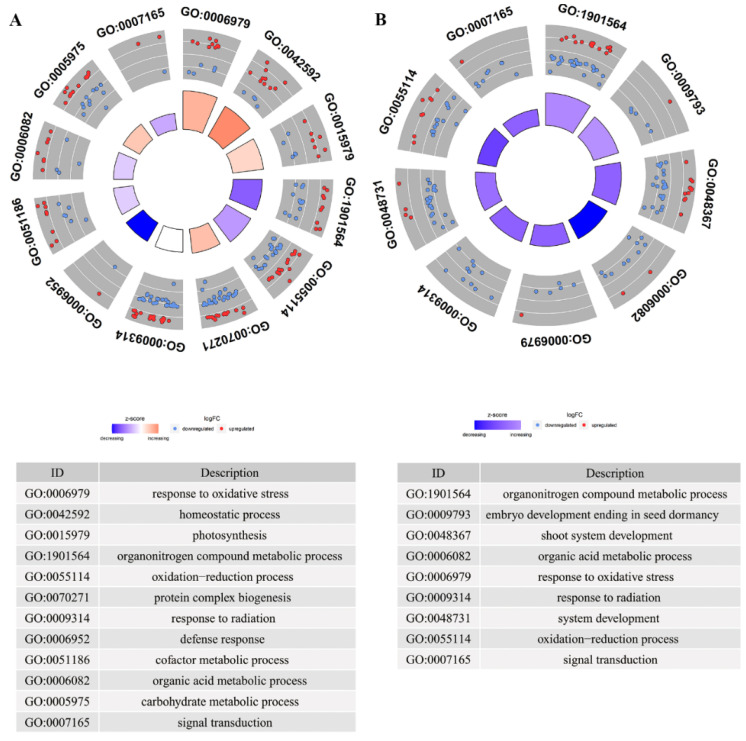
The circle diagram shows the biological process of the significant impact of space flight stress memory on offspring rice. Dots indicate DAPs, red indicates upregulated proteins, and blue indicates downregulated proteins. (**A**) represents the different metabolites at the TLS; (**B**) represents the DAPs at the TS; TLS and TS stand for three-leaf stage and tillering stage, respectively.

In order to understand the impact of spaceflight on the BPs of offspring rice, we used Gene Ontology to annotate the DAPs. GO items with *p* < 0.05 were considered to be a biological process (BP) that significantly changes. In total, 36 BPs were significantly enriched in TLS (Table S3) and 26 BPs were significantly enriched in TS (Table S4). The BPs that were significantly enriched in TLS mainly included BPs related to stress response, protein processing, and signal transduction, while in TS, they mainly included stress response, RNA localization, embryonic development, and organic progeny. Although there were differences in the BPs of the two different growth and development stages, these significantly changed BPs would help us further explain the biological effects of spaceflight in the different growth and development stages of offspring rice. Therefore, we used circle diagrams to describe the changes in proteins involved in these BPs. Response to oxidative stress, oxidation–reduction process, and defense response were significantly enriched in TLS and TS, but their protein abundance patterns were different (Figure 3). These results suggested that the offspring rice retained the ability to respond to spaceflight, which may be induced by oxidative stress, but the offspring rice had different response mechanisms to spaceflight at two different growth stages. In addition, response to radiation was enriched in TLS and TS, but it was interesting that the proteins involved in this BP were all downregulated during TS (Figure 3). This again shows that offspring rice still retain the ability to respond to spaceflight. It was worth noting that the signal transduction process was also enriched, which played an important role in regulating the response of plants to abiotic stress (Figure 3). In order to explore whether these biological pathways have mutual regulation, we used the chord diagram to create the relationship between them. We noticed that there was an interaction between redox reaction, oxidative stress response, and response to radiation and signal transduction (Figure S2). In addition, redox reactions and oxidative stress responses also interacted with cofactor metabolic processes and protein complex biosynthesis (Figure S2A).

We further used the KEGG database to determine whether these differential proteins were enriched in specific metabolic functions. In TLS, 94 DAPs were enriched in 74 KEGG pathways. In TS, 88 DAPs were enriched in 74 KEEG pathways. The pathways enriched in TLS and TS involved amino acid metabolism, energy metabolism, fatty acid metabolism, DNA and RNA metabolism, secondary metabolite metabolism, and protein synthesis and processing. Linking pathways with protein changes allow to understand the impact of spaceflight on these pathways to the greatest extent, so we used circle heat maps to visualize the 12 pathways with the most significant changes. In TLS, we noticed that the DAPs involved in ribosomes were downregulated, and most of the proteins involved in protein processing in the endoplasmic reticulum were downregulated (Figure S3A). The proteins involved in starch and sucrose metabolism, phenylpropanoid biosynthesis, carbon metabolism, biosynthesis of amino acids, glycolysis/gluconeogenesis were upregulated (Figure S3A), and most of the proteins involved in photosynthesis were upregulated (Figure S3A). In TS, the DAPs involved in ribosome, cysteine and methionine metabolism, and endocytosis were downregulated, and the majority of proteins involved in oxidative phosphorylation and biosynthesis of amino acids were upregulated (Figure S3B).

### 2.4. MRNA Expression Validation

**Figure 4:**
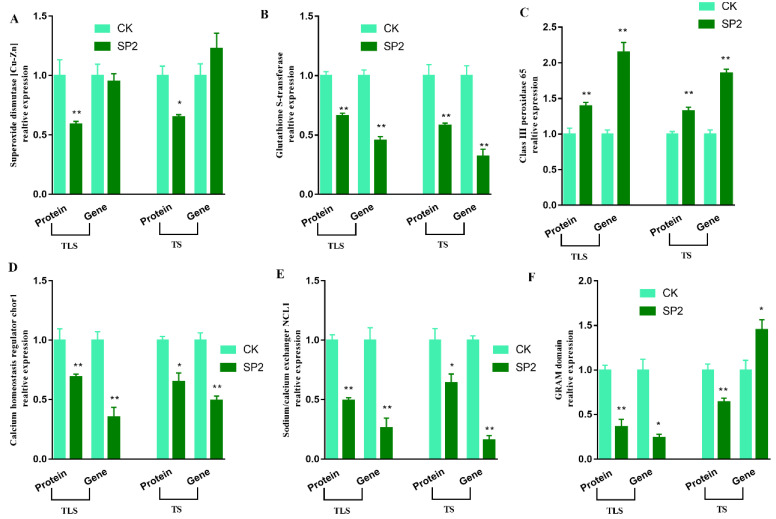
Comparison between protein abundance and mRNA level. The protein abundance in ground treatment and after spaceflight treatment leaves of offspring rice at three-leaf stage (TLS) and tillering stage (TS) were assessed by ITRAQ-based quantitative proteome analysis. Transcript abundance at two development stages (TLP, TS) was determined by quantitative RT-PCR and normalized against the *ACTIN* gene. The cyan column represents the control group; the dark slate gray column represents treatment group. *superoxide dismutase [Cu-Zn]* (**A**), *glutathione transferase*(**B**), *sodium/calcium exchanger NCL1* (**C**), *calcium homeostasis regulator chor1* (**D**), *GRAM domain* (**E**), *Class III peroxidase 65* (**F**). The means and standard error values from three independent samples are shown (means ± SE; *n* = 3). * and ** indicate significant difference at *p* < 0.05 and <0.01 by Tukey’s test, respectively.

**Figure 5:**
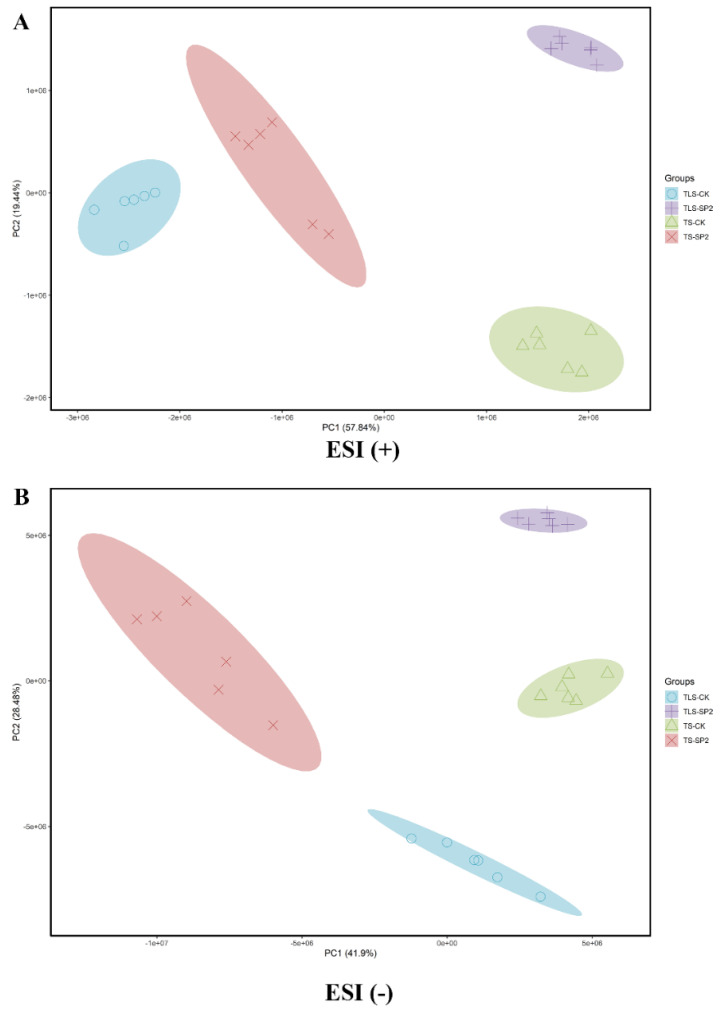
PCA score map of different rice sample groups based on UHPLC-QTOF/MS data (six biological replicates). (**A**) Positive ion mode, (**B**) negative ion mode; TLS and TS represent three leaf period and tillering stage. CK represents control group. SP2 represents the offspring of rice after spaceflight.

RT-qPCR analysis was applied to evaluate whether protein abundances were consistent with transcript levels. Six proteins, superoxide dismutase [Cu-Zn] (Cu-Zn SOD), glutathione transferase, sodium/calcium exchanger NCL1, calcium homeostasis regulator chor1, GRAM domain, Class III peroxidase 65, which showed the same expression abundance trend in the TLS and TS, were selected for transcriptional analysis. In TLS, the gene expression patterns of *glutathione transferase*, *Class III peroxidase 65*, *GRAM domain*, *sodium/calcium exchanger NCL1*, *calcium homeostasis regulator chor1* were consistent with the protein expression profile (Figure 4), which showed the reliability of iTRAQ results. In addition, we observed that the polar shadow expression of *Cu-Zn SOD* and *GRAM domain* was inconsistent with protein expression during TS (Figure 5), which indicated that the effect of mRNA expression level on protein expression abundance was limited.

### 2.5. Spaceflight Changes the Metabolic Response Profile of Offspring Rice

Proteomic studies have proved that spaceflight caused the offspring of rice to respond. However, the realization of protein function should ultimately be reflected at the metabolic level. Therefore, we used Liquid Chromatography Mass Spectrometry (LC-MS) to conduct non-targeted metabolomics studies on offspring rice. In order to evaluate the impact of stress memory on rice at the metabolic level, we used principal components to analyze the distribution of metabolites in the control and treatment groups at different periods (Figure 5). The control group and the treatment group were separated from each other, and the metabolites in different periods were also separated from each other (Figure 5). This indicated that the effect of spaceflight on rice seeds continued to the F2 generation, and different response patterns appeared in different growth and development stages of the F2 generation. At the same time, the mutual aggregation of biological replicates in each group in Figure 4 indicated the reliability and repeatability of metabolomics data.

Hierarchical clustering was used to further analyze the effect of space flight stress memory on metabolites in different growth stages of offspring rice (Figure S4). The results showed that the treatment group and the control group were separated from each other, which indicated that the spaceflight stress memory reshaped the metabolic level of the offspring rice (Figure S4). In addition, the change patterns of metabolites in TLS and TS were different (Figure S4), which indicated that there were differences in the response mechanisms of offspring rice to spaceflight stress and memory in different growth and development stages.

To further evaluate the correlation between proteomics and metabolomics, we used the R language psych to analyze the correlation between differential proteins and differential metabolites. We believe that differential proteins and differential metabolites with correlation coefficients ≥ 0.8 and *p* values ≤ 0.01 are significantly related. Next, we used Cytoscape 3.9.0 to visualize the network (Figure S5). Our results show that TLS and TS are metabolites and proteins show a correlation. It is worth noting that the number of metabolites is always less than that of proteins, which shows that the changes in metabolites are jointly regulated by multiple proteins. In addition, there are 17 metabolites that are related to the proteome in TLS and TS, and among them are compounds involved in amino acid metabolism and sugar metabolism (Figure S5).

### 2.6. Analysis of Characteristic Metabolites through Multivariate Statistical Analysis

**Figure 6:**
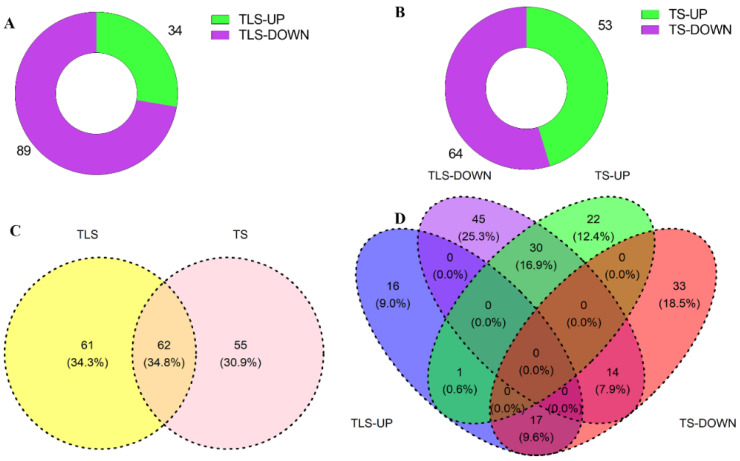
Statistical analysis of differential metabolism of spaceflight stress memory in different growth stages of rice. (**A**) represents the different metabolites at the TLS; (**B**) represents the different metabolites at the TS; (**C**) represents the Venn diagram of the different metabolites at the TLS and the TS; (**D**) represents the Venn diagram of the increase or decrease in the concentration at the TLS and the TS. TLS and TS stand for three-leaf stage and tillering stage, respectively.

To further evaluate the validity of the data, we conducted partial least squares discriminant analysis (PLS-DA). The results showed analytically similar results to PCA (Figure S4). We defined the metabolites with VIP > 1 foldchange ≥ 1.2 or ≤0.83, and *p* value ≤ 0.05 as differential metabolites (DEMs). A total of 123 metabolites changed significantly during the TLS (Table S5), and the concentrations of 34 and 89 metabolites increased and decreased, respectively (Figure 6A). In addition, a total of 117 metabolites changed significantly during the TS (Table S6), among which 53 were increased and 64 decreased (Figure 6B). In order to further evaluate the influence of spaceflight on the metabolites of TLS and TS in the two growth stages, we used Venn diagrams to evaluate the change patterns of the different metabolites in two different growth and development stages (Figure 6C,D). In total, 62 metabolites were shared in two different growth and development stages (Figure 6C), of which 15 metabolites have the same change pattern at two different growth and development stages, while 47 metabolites change patterns were opposite (Figure 6D). We also noticed that 61 and 55 metabolites have unique changes in TLS and TS, respectively (Figure 6C).

Hierarchical clustering was used to further analyze the effect of spaceflight on metabolites in different growth stages of offspring rice (Figure S5). The results showed that the treatment group and the control group were separated from each other, which indicated that the spaceflight reshaped the metabolic level of the offspring rice (Figure S5). In addition, the change patterns of metabolites in TLS and TS were different (Figure S5), which indicated that there were differences in the response mechanisms of offspring rice to spaceflight in different growth and development stages.

To further evaluate the correlation between proteomics and metabolomics, we used the psych package to analyze the correlation between DAPs and DEMs. We believe that DAPs and DEMs with correlation coefficients ≥ 0.8 and *p* values ≤ 0.01 are significantly related. Next, we used Cytoscape 3.9.0 to visualize the network (Figure S6). Our results show that TLS and TS are metabolites and proteins that show a correlation. It is worth noting that the number of metabolites is always less than that of proteins, which shows that the changes in metabolites are jointly regulated by multiple proteins. In addition, there are 17 metabolites that are related to the proteome in TLS and TS, and among them are compounds involved in amino acid metabolism and sugar metabolism (Figure S6).

### 2.7. Spaceflight Reshapes the Metabolic Level of Offspring Rice

**Figure 7:**
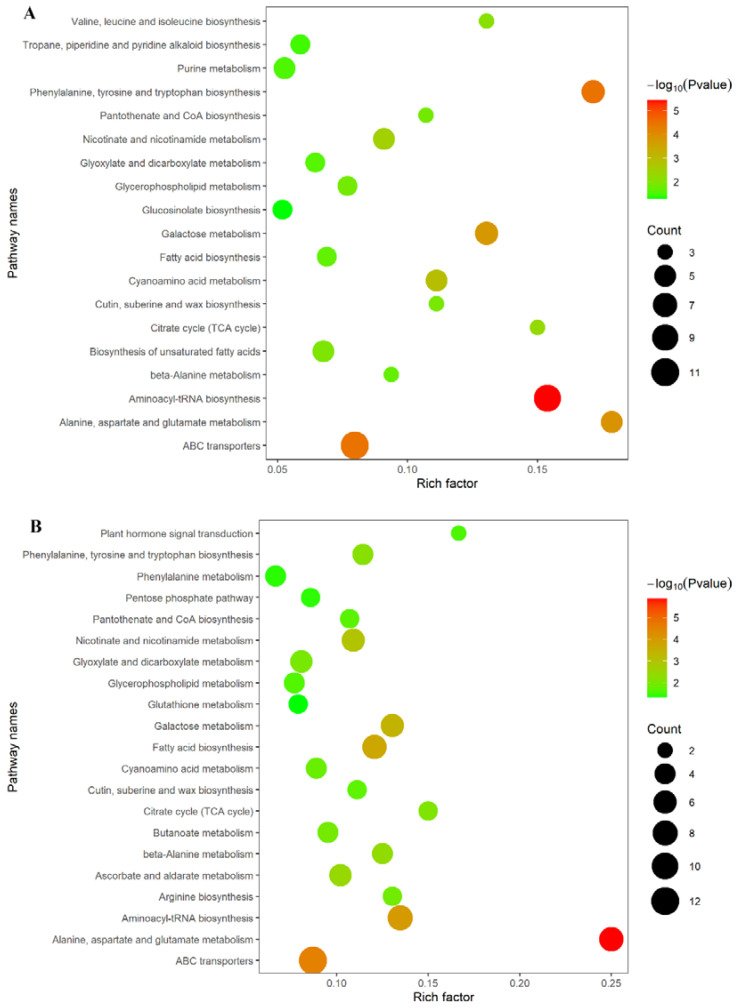
Analysis of metabolic pathways of TLS and TS. Results were from the analyses using the MetaboAnalyst 4.0 software. Every circle represents a metabolic pathway, with red color indicating higher impact and yellow color indicating lower impact. The size of the circle represents the number of DEMs participating in the pathway. (**A**) Rice samples at the TLS; (**B**) rice samples at the TS.

In order to further explore the impact of spaceflight on offspring rice at the metabolic level at different growth and development stages, we used the KEGG database to evaluate the metabolic pathways involved in different metabolites. Different metabolites in TLS and TS were enriched in 62 and 66 KEGG pathways. We used bubble graphs to show the metabolic pathways with *p* < 0.05, which were considered to be metabolic pathways that have a significant impact on spaceflight (Figure 7). There were 19 metabolite pathways that were significantly enriched in TLS (Figure 7A) and 21 metabolic pathways were enriched in TS (Figure 7B). Interestingly, there were 14 metabolic pathways that were significantly enriched in two different growth stages. These shared metabolite pathways affected the plant’s amino acid metabolism, genetic information processing, environmental information processing, lipid metabolism, energy metabolism, sugar metabolism, cofactors, and vitamin metabolism.

### 2.8. Metabolic Network Reveals the Response Mechanism of Offspring Rice Metabolites to Spaceflight

**Figure 8:**
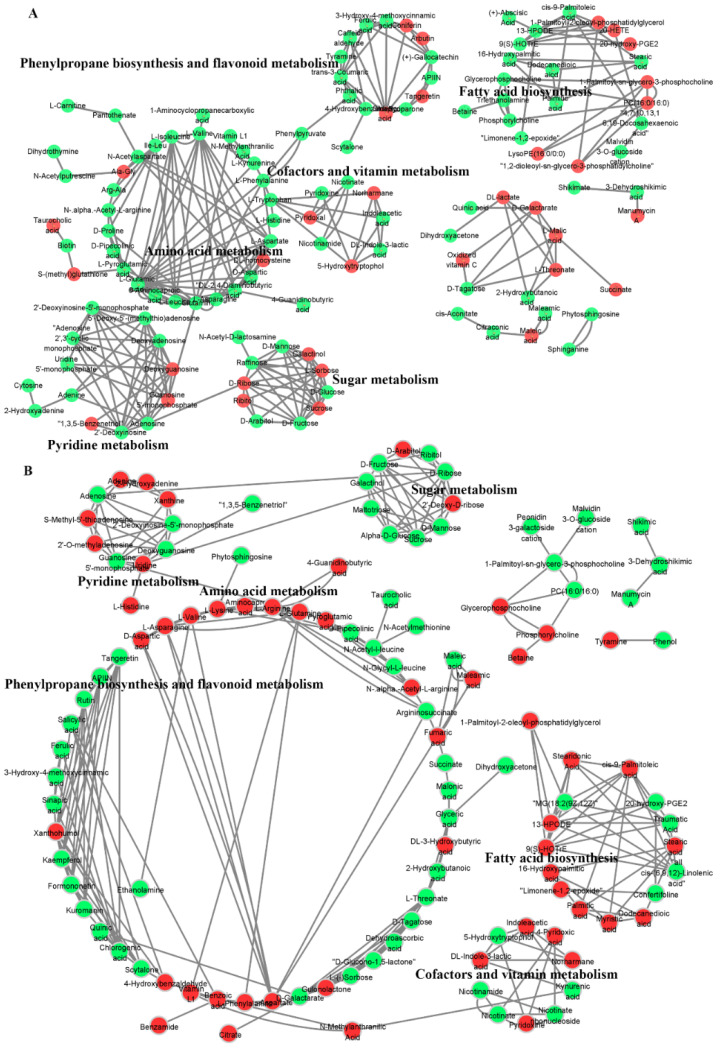
The metabolite–metabolite interaction network of significantly different metabolites that participated in KEGG in rice subjected to the TLS (**A**) and TS (**B**) experiments was developed using the cytoscape software. Red represents increased metabolites (SP2 vs. CK) and green represents decreased metabolites (SP2 vs. CK).

MetaMapp 2020 (http://metamapp.fiehnlab.ucdavis.edu/ocpu/library/MetaMapp2020/www/, accessed on 17 March 2022) was used to integrate biochemical pathways and chemical relationships and connect different metabolic pathways through key metabolites in series to reveal the inherent biological effects. In order to obtain key information about the offspring rice responses to spaceflight at the metabolic level, we used MetaMapp to analyze the interaction of different metabolites. The PubChem database was used to obtain the structural information of the metabolites, the KEGG database was used to obtain the KEGG codes of the metabolites, the interaction analysis was performed in the MetaMapp online database, and the interaction network was visualized using Cytoscape 3.9.0 (Figure 8). The two different growth and development stages of TLS and TS have different metabolite interaction networks, which meant that rice after spaceflight have different response mechanisms to spaceflight in different growth and development stages of the offspring. Compared with TS, the metabolites of offspring rice at TLS had a more complex metabolite interaction network (Figure 8). Obviously, in two different growth and development stages, amino acid metabolism, pyridine metabolism, sugar metabolism, cofactor and vitamin metabolism, phenylpropane biosynthesis and flavonoid metabolism formed a metabolic network through key metabolites, and amino acid metabolism was at the center of this network (Figure 8). In the TLS, L-phenylalanine, phenylpyruvate, and phthalic acid were key metabolites that interacted with amino acid metabolism and phenylpropane biosynthesis and flavonoid metabolism (Figure 8A). L-tryptophan, DL-indole-3-lactic acid, indoleacetic acid, and norharmane were key metabolites that interacted with amino acid metabolism and cofactors and vitamin metabolism (Figure 8A). L-glutamine and guanosine 5′-monophosphate were key metabolites of amino acid metabolism and pyridine metabolism (Figure 8A). Adenosine and D-ribose connect pyridine metabolism with sugar metabolism (Figure 8A). During the TS, amino acid metabolism, pyridine metabolism, and sugar metabolism also interacted through L-glutamine, guanosine 5′-monophosphate, adenosine, and D-ribose (Figure 8B). The difference from the TLS was that in the TS, amino acid metabolism and phenylpropane biosynthesis and flavonoid metabolism interact through L-phenylalanine and benzoic acid (Figure 8B). In addition, amino acid metabolism and cofactors and vitamin metabolism interacted through L-phenylalanine, benzoic acid, vitamin L1 (Figure 8B). Obviously, the key metabolites of these network nodes play an important role in the response of offspring rice to spaceflight.

## 3. Discussion

Our previous studies demonstrated that spaceflight reshaped the metabolic network of parental rice. Oxidative stress was the main factor that causes rice to respond to spaceflight. Rice adapted to the effects of spaceflight by regulating amino acid metabolism, protein synthesis and degradation, protein processing, energy metabolism, and sugar metabolism [11,12,13]. At present, studies have confirmed that the effects of spaceflight on organisms were heritable, which was caused by the instability of the organism’s genome due to radiation and microgravity. Research on how progeny plants respond to spaceflight were rarely reported. Therefore, in this study, we used metabolomics and proteomics to reveal the response mode of offspring rice to spaceflight. This provided not only a new perspective for the systematic interpretation of the biological effects of spaceflight, but also provides a new perspective for revealing how plant offspring respond to spaceflight.

### 3.1. Offspring Rice Response to Spaceflight Relies on Redox Modulation and Signal Transduction

Abiotic stress greatly affected plant growth and cell redox state. Studies have shown that high temperature, cold, drought, aluminum poisoning, organic pollution, and other abiotic stresses can induce the production of reactive oxygen species (ROS) in plants [17,18,19,20,21]. Our previous work also reported that spaceflight caused an increase in ROS content in the parent rice [11,12]. Too low or too high ROS levels can impair plant growth and development and keeping ROS levels within an appropriate range can promote plant health [22]. However, ROS, as a signal molecule, triggered the signal transduction pathway to respond to abiotic stress, which was a necessary process for endowing plants with resistance to abiotic stress [23]. In this study, the content of H_2_O_2_ in the SP2 group was significantly increased in the TLS and TS stages (Figure 1). At the same time, the activities of MDA, EL, SSC, and antioxidant enzymes that characterized the level of oxidative stress also changed significantly (Figure 1). These results suggested changes in the redox state of offspring rice. The H_2_O_2_ signaling pathway led to the accumulation of protective agents, which prevented changes in the redox state of the cell from oxidative damage [24]. Furthermore, we classified the functions of proteins and look forward to confirming the existence of stress responses at the protein level. There was a total of 18 proteins involved in the redox reaction at the TLS (Figure S7A), including thioredoxin family proteins, peroxidase family proteins, and superoxide dismutase [Cu-Zn] abundance changes. A total of 13 proteins were involved in the redox reaction during the TS (Figure S7A). They mainly included glutathione S-transferase family proteins, peroxidase family proteins, and superoxide dismutase. It was well known that peroxidase and superoxide dismutase were very important for maintaining the redox state balance in cells under abiotic stress. [25,26]. Glutathione S-transferase and thioredoxin family proteins were considered to be ROS sensors that triggered various signal cascades and were key factors that caused transcription/translation. In our research, significant changes in these types of proteins have also been observed.

Studies have confirmed that oxidative stress signals can activate downstream signal cascades [22]. Our results showed that spaceflight caused the stress response of offspring rice (Figure 1 and Figure S7), and this response was triggered by oxidative stress. In this study, there were changes in the abundance of proteins involved in calcium, G protein, and kinase-mediated signaling pathways (Figure S7B). The small GTP binding protein was converted from the inactive form of GDP binding to the active form of GTP binding under the activation of the upstream signal (ROS), which led to conformational changes in the downstream effector binding region, thereby realizing signal transduction [27]. After that, the intrinsic GTPase converted the GTP-bound form to the GDP-bound form, and then released the bound downstream effectors. In this way, a cycle of activation and inactivation was achieved, and the small GTP binding protein acted as a molecular switch, transducing upstream signals to downstream effectors in the process. Guanine Nucleotide Exchange Factor (GEF) catalyzed the release of bound GDP to form an active small GTP binding protein that binds to GTP [28]. The results of this study showed that there were significant changes in the protein abundance of two G protein signaling pathways (small GTP-binding protein RAB5B and guanine nucleotide-exchange protein GEP2) (Figure S7B). This indicated that G protein signaling was involved in the response of offspring rice to spaceflight. In addition, studies have proved that small GTP-binding protein RAB played an important role in vesicle transport, pollen tube and root hair development, Ca^2+^ signal, and hormone signal crosstalk [28].

Ca^2+^ signal has been confirmed to be involved in abiotic stresses such as drought, cold, and dehydration [29,30]. In this study, the abundance of IQ calmodulin-binding motif, sodium/calcium exchanger NCL1, protein IQ-DOMAIN 31, calcium homeostasis regulator chor1, calmodulin binding protein, and EF hand family protein changed significantly (Figure S7B). Calcium-binding protein can reduce the concentration of calcium ions in cells, thereby reducing the concentration of ROS in cells [30], which may also be consistent with the ROS results in this study (Figure S7B). These proteins involved in the calcium ion signal transduction pathway have also changed in contemporary rice plants after spaceflight [11], which showed that calcium ion signals played an important role in regulating spaceflight and can be remembered in offspring plants.

Small GTP-binding protein RAB and calcium binding protein further interacted with plant hormones IAA, ABA, GA, BR, and regulated stress signals [28,31,32]. This study identified changes in ABA and IAA levels (Figure S6). At the same time, significant changes in the expression abundance of proteins involved in the metabolism of ABA (9-cis-epoxycarotenoid dioxygenase 1, GRAM domain, and GEM protein 1), IAA (ARP1), and JA (lipoxygenase, 12-oxophytodienoate reductase 7) (Figure S7B) were also identified. These changes were also found in our previous proteomic studies [11].

### 3.2. Genetic Material Metabolism and Protein Reorganization Are the Key Processes of Offspring Rice Coping with Spaceflight

DNA double-strand break (DSB) produced by ionizing radiation during spaceflight was considered to be the main factor posing a serious threat to genome stability [33], and genome instability has been proven to be a key factor for plants to have exaggerated memories of abiotic stress [34]. Plants ensured the stability of their genome by removing DNA damage and reconstructing the original genetic information, thereby ensuring faithful replication, correct organization, and transcription [35]. In our study, the abundance of four proteins involved in the DNA repair process changed significantly (cell cycle checkpoint protein RAD17, chromatin assembly factor 1 subunit FSM, DNA mismatch repair protein MLH1, DNA polymerase delta subunit 3, and replication factor A1) (Figure S8A). Cell cycle checkpoint protein RAD17 was involved in the regulation of DNA repair related to non-homologous double-strand break (DSB) repair, but it was involved in fast but not slow DSB repair mechanisms [36]. This protein was upregulated in TLS, which indicated that the DNA damage process caused by endogenous ROS was intensified at this time. Chromatin assembly factor 1 subunit FSM was the p150 subunit of Chromatin Assembly Factor-1 (CAF-1). FSM was closely related to specific cell cycle stages. FSM was necessary for plants to maintain flat and small shoot apical meristems (SAM). Rice that silenced this protein exhibited seedling growth defects and died during the vegetative stage. In our study, FSM had different expression patterns in TLS and TS, which indicated that TLS was more active in cell division than TS. Interestingly, different subunits of histones were all upregulated in our study (Figure S8A). Histones played a central role in transcriptional regulation, DNA repair, DNA replication, and chromosome stability [37]. In addition, histones also participate in SA-mediated signaling pathways [37]. Therefore, we inferred that the instability of the genome in the offspring rice after spaceflight would affect the transcriptional regulation of its genes. In addition, DNA polymerase delta subunit 3 is also involved in the DNA methylation process and plays an important role in the base excision repair process of exogenous DNA methylation damage [38]. In this study, DNA polymerase delta subunit 3 underwent significant changes in both TLS and TS, so the DNA methylation process may have changed in this study.

Transcription factor (TF) played an important role in plant stress response by interacting with cis-acting elements in the promoter and then regulating the expression of its target genes [39]. Our previous studies have also confirmed that spaceflight can cause changes in TF in parent rice [12]. In this study, we also found some changes in transcription factors (Figure S7B). Among them, the abundance of four transcription factor proteins changed significantly in TLS and TS. In addition to MAR-binding filament protein 1, the abundance of the other three proteins (PHD finger protein ALFIN 2, CBFD_NFYB_HMF domain-containing protein, and homeobox protein knotted-1) had the same trend in these two periods. PHD finger protein ALFIN 2 was a plant-specific gene ALFIN family protein, which played an important role in root growth and response to abiotic stress. ALFIN protein can improve the survival rate of plants under drought stress, reduce the content of malondialdehyde (MDA), and reduce water loss [40]. Therefore, the increase in MDA content in this study (Figure 1) may be related to the decrease in the abundance of this protein. CBFD_NFYB_HMF domain-containing protein was one of the subunits of transcription factors with the CCAAT motif. Many studies have clarified that the transcription factor subunits of the CCAAT motif played a role in the gametogenesis of the complex, embryogenesis, seed development, flowering time regulation, primary root elongation, ABA signaling, drought resistance, endoplasmic reticulum stress response, and hypocotyl elongation [41,42,43]. Inhibition of GA activity by homeobox protein knotted-1 (KNOTTED1) transcription factor was a key component of meristem function. The KNOTTED1 and GA regulatory modules were transferred from the meristem to the leaves to change the morphology of the plant. Therefore, the differential abundance of this protein implied that spaceflight may change the morphology of rice leaves. In addition, we also identified that the abundance of other transcriptional regulators changed significantly in TLS and TS, respectively.

For most eukaryotic genes, introns must be spliced from the precursor mRNA (pre-mRNA) for various processing to produce mature mRNA for protein translation [44,45] Therefore, precursor mRNA processing was critical to gene expression. In this study, changes in protein abundance related to RAN processing (RAN shearing, RNA unwinding, RNA transport) were identified (Figure S8C). This implied that the protein synthesis process of TLS and TS was affected in this study.

Notably, the expression of ribosomal 30S subunit and ribosomal 50S subunit was upregulated in this study (Figure S8C), while the expression abundance of ribosomal 40S subunit and ribosomal 60S subunit was downregulated. Silent expression of ribosomal 30S subunit and ribosomal 50S subunit occurs [46]. Therefore, we concluded that spaceflight may significantly change the photosynthetic response of offspring rice. The ribosomal 40S subunit and the ribosomal 60S subunit were the only places where cells synthesized most proteins. Downregulation of the protein abundance of these two subunits would result in a decrease in the rate of protein synthesis. Studies have shown that changes in the magnetic field can lead to changes in the structure of the nucleolus of plant cells, affecting the formation of ribosomes [10]. Therefore, the decrease in the abundance of the 40S subunit and the ribosomal 60S subunit in this study is due to changes in the magnetic field during spaceflight. Moreover, our results also showed the downregulation of protein translation initiation factor abundance during TLS and TS (Figure S6C), which again showed that spaceflight reduces the rate of protein synthesis. Our previous work also showed that spaceflight has caused a decrease in the synthesis rate of contemporary rice protein [11,12]. Obviously, the decrease in rice protein synthesis rate caused by spaceflight was heritable. In addition, we also identified that protein abundance involved in protein processing (Figure S8C), degradation, and ubiquitination also occurred. These pathways were very important for plants to maintain the correct protein structure under abiotic stress.

The above results indicated that the oxidative stress generated by spaceflight has caused the instability of the offspring rice genome, leading to changes in the process of gene transcription and post-transcriptional modification. At the same time, the protein synthesis rate in the progeny rice was reduced, and the subsequent protein processing and degradation processes were affected. These changes may cause the metabolic rearrangement of offspring rice to adapt to the impact of spaceflight.

### 3.3. Spaceflight Reconstructs the Metabolic Network of Offspring Rice

Spaceflight induced changes in rice protein levels in offspring, which aroused our attention towards metabolic networks. We used MetaMapp to construct the metabolic network of spatial progeny rice, hoping to reveal how the progeny rice can adapt to spaceflight by reshaping the metabolic network (Figure 7). Amino acid metabolism, sugar metabolism, cofactor and vitamin metabolism, purine metabolism, phenylpropane biosynthesis and flavonoid metabolism, and fatty acid metabolism constituted the metabolic network of offspring rice in response to spaceflight (Figure 7).

According to reports, as the basic elements of protein, amino acids can be used as effective antioxidants and accumulate in plants under various abiotic stresses [47]. Here, we identified changes in the concentration of several amino acids, as well as changes in the abundance of proteins involved in amino acid metabolism (Figure S9). We noticed that in the three-leaf stage, except for the increase in the concentration of DL-homocysteine and L-threonate, the concentration of other amino acids all showed a decreasing trend (Figure S9A). In TLS, except for L-threonate, the concentration of other amino acids showed an increasing trend, which indicated that spaceflight has different influence modes on the amino acid metabolism of offspring rice.

Sugar has been characterized as an important compound that plants responded to abiotic stress [48]. Soluble sugars and sugar alcohols also acted as compatible solutes [49] and were responsible for regulating osmotic balance and protecting proteins and other macromolecules from damage caused by stress [50]. Moreover, sugar can also regulate the expression of various genes involved in photosynthesis and respiration, so that plants can adapt to environmental stress [51]. Here, various sugars such as D-mannose, L-sorbose, raffinose, sucrose, D-glucose, D-fructose, D-ribose, and D-tagatose were significantly changed in the two different growth periods of the offspring rice (Figure S10). This was the same as the change in the soluble sugar content in our Figure 1. Glucose regulates effector genes through the Target of Rapamycin (TOR) kinase signaling cascade, which regulated glycolysis and the metabolism of stress-responsive sugars (such as sucrose, raffinose, and starch) [52]. Glucose was converted to glucose 6-phosphate (G6P) through HXK activity, and was further used in the synthesis of polyols, including inositol, sorbitol, and mannitol. In our study, the content of glucose decreased during two different growth periods (Figure S10), which may be one of the reasons for the decrease in other sugars. In addition, glucose was the main product of photosynthesis, and the consumption of glucose indicated that photosynthesis may be reduced [53]. In our previous study, we also confirmed the changes in these sugars in parent rice after spaceflight [13]. These results indicated that sugar metabolism was also the main way to respond to spaceflight and can be remembered.

Under most abiotic stress conditions, leaf starch content decreases in response to abiotic stress, and starch degradation in response to stress was usually related to an increase in tolerance [54]. Starch synthase was involved in the biosynthesis of starch. In this study, their protein abundance was reduced (Figure S10B), which indicated that the biosynthesis of starch was inhibited at this time. In addition, we noticed that the abundance of beta-amylase was also reduced (Figure S10B). Studies have confirmed that transient starch was degraded during drought stress to maintain proline biosynthesis [55]. Therefore, under most abiotic stresses, plants adapted to stress conditions by increasing the expression or activity of amylase [54]. Our research results were slightly different from those reported in the literature, which may be due to the effect of spaceflight being reduced as the rice grew and developed. This also explained why starch synthase and beta-amylase were not differentially expressed in TS. These results suggested that sugar metabolism can be remembered, so that changes in sugar metabolism pathways can still be observed in offspring rice.

Our previous work has observed the effects of spaceflight on the metabolism of cofactors and vitamins in the parent rice [13]. Nicotinamide can induce plant stress and defense-related metabolic processes. In addition, it was also related to oxidative damage and DNA strand breaks under stress conditions [56]. In this study, the content of nicotinamide and nicotinate was reduced, and the reduction in TS was more than that in TLS (Figure S11). This may also be one of the reasons for the different abundance of protein profiles in the two different growth phases. Furthermore, nicotinamide and nicotinate can significantly affect the epigenetic process, which was the main reason for stress to be remembered [56]. Therefore, in this study, the performance of offspring rice may be related to epigenetic effects. Nicotinate phosphoribosyltransferase was an important enzyme in the NAD salvage pathway, using nicotinate as a substrate. It has been shown that mutations in the gene encoding NaPRTase (OsNaPRT1) in rice led to increasing levels of nicotinamide and nicotinate [57]. In this study, nicotinate phosphoribosyltransferase was upregulated in TS (Figure S11B), which may be the reason for the decrease in nicotinamide and nicotinate. Pyridoxine and pyridoxal were two forms of vitamin B6. Vitamin B6 played an important role as a cofactor in a wide range of biochemical reactions. In addition, it was also related to oxidative damage caused by oxidative stress [58]. Vitamin B6 has been proven to be an excellent antioxidant in vitro. Current studies have confirmed that vitamin B6 indirectly achieved plant defense against abiotic stress by supporting peroxidase activity instead of directly eliminating ROS. The defense pathway affected peroxidase through the association of cysteine and vitamin B6 biosynthesis [59]. Our results showed that pyridoxal only accumulates in TLS. Pyridoxine was reduced in TLS but was accumulated in TS (Figure S11B). This was somewhat different from the expression patterns of these two compounds in contemporary rice after spaceflight as we reported earlier [13]. Although offspring rice inherits the effects of spaceflight, it responds in a different way than contemporary rice. We also noticed the changes in vitamin L1 and oxidized vitamin C (Figure S11B). Vitamin L1 was the main precursor for the synthesis of auxin, and it played an important role in root gravity by regulating the polarity and relocation of PIN-FORMED protein. Therefore, the changes in the concentration of auxin in this study may be related to the changes in the concentration of vitamin L1. Oxidized vitamin C was a compound that has been proven to have an antioxidant effect and was known for scavenging ROS in the body. Here, oxidized vitamin C was accumulated in the TLS stage (Figure S11A), which indicated that the spaceflight has an oxidative stress effect on the offspring rice at this time, which also confirmed that our results in Fig1 were accurate. Our results suggested that spaceflight reshaped the vitamin metabolism of offspring rice, especially the B vitamins. More interestingly, these vitamins can respond to the oxidative stress effects of abiotic stress. Therefore, we can once again confirm that the spaceflight produced an oxidative stress effect on offspring rice, and this effect continued from TLS to TS.

### 3.4. Proteins That Were Remembered across Generations under the Stress of Spaceflight

To further understand the genetic mechanism of progeny rice for space flight, here we conducted a comprehensive analysis of previous reports and current results [11]. In TLS, 18 proteins were continuously affected by spaceflight, while in TS, 16 proteins are remembered. It is worth noting that only the abundance change pattern of 12-oxophytodienoate reductase 7 protein was opposite between the parent and the offspring, and the other proteins show the same change pattern (Table S7). These memory proteins were mainly involved in redox state, signal transduction, nucleic acid metabolism, protein synthesis, and plant hormone metabolism processes (Table S7). Obviously, spaceflight may continue to affect this biological process and cause the offspring of rice to respond and adapt to spaceflight by rebuilding their own metabolic network.

## 4. Materials and Methods

### 4.1. Plant Preparation and Growth Conditions

The experiment was part of the SJ-10 space project, which was launched from the Jiuquan Satellite Launch Center in April 2016. The spaceflight parameters were consistent with what we reported earlier [11,13]. According to our experimental design, we randomly selected 30 plants from the planted Dongnong416 parent plants for parental experiments and harvested these 30 plants for planting. We mixed the seeds of these 30 rice plants and randomly selected 400 seeds for planting to obtain F2 generation plants. The control sample was also obtained using the same method. All the seeds were planted in large fields in Wuchang City, Heilongjiang Province. Among the F2 generation plants, we randomly collected the rice leaves in the TLS and TS periods, respectively, for the following experiments. SP2 was used to represent the F2 generation plant of the rice seed after spaceflight, and CK to represent the F2 generation plant of the rice seed without spaceflight.

### 4.2. Determination of Physiological Indicators

We used the methods described by Yu et al. [60] and Heath [61] to determine H_2_O_2_ and MDA, respectively. In short, about 200 mg of rice leaves were homogenized (0.1% *w*/*v*) in 2 mL of trichloroacetic acid and centrifuged at 4 °C to obtain the supernatant for use. Then, 0.75 mL pH = 0.7 potassium phosphate buffer and 1 mL potassium iodide (1 M) were added to 0.25 mL supernatant and mixed, the absorbance was read at 390 nm, and the H_2_O_2_ content was calculated. For MDA, we added 1.5 mL of thiobarbituric acid to 0.5 mL of supernatant, and after reacting at 90 °C for 20 min, the reaction was terminated on ice. Finally, the absorbance at 535 and 600 nm was read to calculate the MDA content.

For the determination of antioxidant enzyme activity, we placed about 0.5 g of rice leaves in 5 mL phosphate buffer (0.1 mol/L, pH 7.4) and homogenized on ice. After fully grinding, the supernatant was collected by centrifugation to obtain the crude enzyme extract for activity analysis. The activity of SOD and APX was measured according to the method described by [62]. The catalase (CAT) activity was measured according to the method described in Aebi [63]. The peroxidase (POD) activity was measured according to the method described by Castillo et al. [64].

We used the method described by LUTTS et al. [65] to determine the electrolyte leakage rate (EL). In total, 0.5 g of rice leaves were placed in 25 mL of deionized water for 3 h (25 °C), and the measured conductivity was N1. Next, they were boiled for 10 min and cooled to 25 °C. The measured conductivity was N2. EL was calculated by the formula EL = N1/N2 × 100%.

For soluble sugars, we used the method described by BAILEY et al. [66] for determination. In short, a 100 mg sample was extracted in 5 mL of 80% ethanol for 30 min (80 °C), and the supernatant was collected by centrifugation. The extraction was repeated 3 times. Then, the anthrone reagent was added to the supernatant for color reaction (95 °C, 20 min). After the reaction, the absorbance was measured at 620 nm to calculate the soluble sugar content.

### 4.3. Protein Identification and Quantification by iTRAQ

The leaves of 5 rice plants were randomly selected to form a single biological replicate. For the proteomic experiment, we conducted 3 biological replicates. Protein extraction, LC-MS analysis, protein identification, and relative quantification were carried out according to the methods we previously reported [11,12]. Simply, the rice leaves were ground into powder in liquid nitrogen, the powdered sample was transferred to a centrifuge tube, and 25 mL of trichloroacetic acid/acetone mixture, and precipitation was carried out at −20 °C for 1 h. Next, it was centrifuged at 10,000 rpm for 45 min, the supernatant was removed, and the pellet was dried. Then the STD buffer (4% SDS, 1 mM DTT, 150 mM Tris HCl pH 8.0) was added to the precipitate and mixed well, boiled in a water bath for 5 min, and centrifuged to dry to obtain the protein. Next, 200 μg of the extracted protein was used for enzymatic hydrolysis to obtain peptides. About 80 μg of peptides were used to label the peptides according to the iTRAQ Reagent-8plex MultiplexKit (AB SCIEX, Framingham, MA, USA) instructions, and then SCX classification was performed. Afterwards, the separation was performed using the Easy nLC HPLC liquid phase system (Thermo Finnigan, San Jose, CA, USA) with nanoliter flow rate. The specific parameters are as follows: mobile phase A is 0.1% formic acid aqueous solution and mobile phase B is 0.1% formic acid acetonitrile aqueous solution (84% for acetonitrile). The loading column is Thermo scientific EASY column (Thermo Finnigan, San Jose, CA, USA, 2 cm × 100 μm × 5 μm-C18), and the analytical column is Thermo scientific EASY column (Thermo Finnigan, San Jose, CA, USA, 75 μm × 100 mm × 3 μm-C18), and the separation process flow rate is 250 nL/min. The following gradient was used for elution: 0–50 min, the linear gradient of liquid B is from 0% to 35%; 50–54 min, the linear gradient of liquid B is from 35 to 100%; Q-Exactive mass spectrometer (Thermo Finnigan, San Jose, CA, USA) was used for mass spectrometry analysis. The analysis time was 60 min, the detection method was positive ion, the precursor ion scan range was 300–1800 *m*/*z*, and the resolution of the primary mass spectrum was 70,000 at *m*/*z* 200. The mass-to-charge ratios of peptides and peptide fragments were collected according to the following method: 10 fragment maps (MS2 scan) were collected after each full scan. MS2 Activation Type was HCD, isolation window was 2 *m*/*z*, MS resolution was 17,500 at *m*/*z* 200, and normalized collision energy was 30 eV. The original data of mass spectrum analysis was RAW file. The RAW file was submitted to the Mascot server through Proteome Discoverer1.4, the established database was selected, and then the database was searched. The Proteome Discoverer1.4 software was used to quantitatively analyze the peptide reported ion peak intensity. The predicted protein was downloaded from the Uniprot database (https://www.uniprot.org/, accessed on 16 August 2020). In order to determine the relative difference in protein abundance, we defined proteins with a fold change rate ≥ 1.2 or ≤0.83 and *p* < 0.05 as differentially abundant proteins (DAPs).

### 4.4. HPLC-MS/MS for Metabolomics

Similar to proteomics, we randomly selected 5 rice leaves to form a single biological replicate and created 6 biological replicates. The metabolites were extracted and identified according to our previously reported methods [13]. In short, about 0.1 g of sample was fully ground in liquid nitrogen, and 1 mL of methanol:acetone:water mixture was added, and extracted under ultrasonic conditions for 60 min (100 w, 4 °C). Next, it was left to settle naturally at −20 °C for 1 h, and then centrifuged to collect the supernatant. The supernatant was vacuum dried and then 100 μL acetonitrile:aqueous solution (1:1, *v*/*v*) was added for subsequent HPLC-MS/MS analysis. The parameters of HPLC and MS have been reported in detail in our previous studies [13]. ProteoWizard 3.0.21229 was used to convert the data mirror, and then the XCMS program was used to perform program peak alignment, retention time correction, and peak area extraction. Metabolite structure identification was carried out by accurate mass matching (<25 ppm) and secondary spectrum matching. Reference material databases were built by Dalian Institute of Chemical Physics and Shanghai Zhongke New Life Biotechnology Co., Ltd. The data was input into the software SIMCA-P 14.1 (Umetrics, Umea, Sweden) for pattern recognition, preprocessed by Pareto-scaling, and then subjected to multi-dimensional statistical analysis. Partial least squares discriminant analysis (PLS-DA) and metabolic pathway analysis were both carried out by MetaboAnalyst 4.0 software (http://www.metaboanalyst.ca/, accessed on 4 November 2021). Principal component analysis (PCA) was undertaken using R software.

### 4.5. Quantitative Real-Time PCR (qRT-PCR) Analysis

The same method as we reported earlier was followed [11,12]. The TaKaRa kit (9767, TaKaRa, Beijing, China) was used to extract total RNA and 1% (*w*/*v*) denaturing agarose gel electrophoresis and Micro Drop (BIO-DL Co. Ltd., Shanghai, China) were used to evaluate the quality and concentration of total RNA. Next, the TaKaRa kit (RR037A, TaKaRa, Beijing, China) was used to reverse transcribe total RNA into cDNA. SYBR Premix Ex Taq II (TaKaRa kit 820A, TaKaRa, Beijing, China) was used to detect gene expression. The relative expression level of each gene was calculated as (2^−ΔΔCT^) [67]. All primers are listed in Table S8. All qRT-PCR were performed with three biological replicates.

### 4.6. Bioinformatics Analysis

Gene Ontology and MAPMAN were used for protein function annotation. Kyoto Encyclopedia of Genes and Genomes (KEGG) (http://www.kegg.jp/, accessed on 7 December 2021) was used to analyze the major metabolic pathways of DAPs and DEMs. We believed that the GO terms and KEGG pathways were significantly enriched when *p* values ≤ 0.05.

## 5. Conclusions

This study investigated the changes in protein and metabolic levels of offspring rice at different growth and development stages caused by spaceflight. Our findings suggest that spaceflight elicits a stress response in offspring rice, and that this stress signal results from a disruption of redox balance in the plant. ROS signaling triggers the crosstalk between calcium ion signaling and hormone signaling, affecting gene transcription and post-transcriptional modification processes, which in turn affects protein synthesis and modification processes. In addition, changes in the magnetic field during spaceflight led to the study of 40S ribosomes and 60S ribosomal subunit abundance was reduced, which was the most important factor leading to the differential abundance of proteins in progeny rice. Furthermore, we demonstrated at the metabolic level that spaceflight remodeled the metabolic network of offspring rice, especially amino acid metabolism, sugar metabolism, vitamin and cofactor metabolism. This study revealed the response mechanism of offspring rice to spaceflight from the perspective of metabolomics and proteomics, and provided basic data for further exploration of the biological effects of spaceflight.

## Data Availability

The authors confirm that data supporting the findings of this study are available in the article and its Supplementary Materials. The raw data is available through direct contact with the corresponding author.

## Supplementary Materials

The following supporting information can be downloaded at: https://www.mdpi.com/article/10.3390/ijms23063390/s1.

## Abbreviations

TLS	Three-leaf stage
TS	Tillering stage
SJ-10	ShiJian-10 retractable satellite
DN416	Dongnong416
SP2	F2 generation space flight group
CK	F2 generation control group
EL	Electrolyte leakage rate
POD	Peroxidase
CAT	Catalase
APX	Ascorbate peroxidase
SOD	Superoxide dismutase
ROS	Reactive oxygen species
SSC	Soluble sugar content
PCA	Principal component analysis
iTRAQ	Isobaric tags for relative and absolute quantification
qRT-PCR	Real-time quantitative PCR
