# Supplementary material for: The Memory of Rice Response to Spaceflight Stress: From the Perspective of Metabolomics and Proteomics
Source: Int J Mol Sci. 2022 Mar 21;23(6):3390. doi: 10.3390/ijms23063390 (PMC8954569; doi:10.3390/ijms23063390)
Supplement: Supplementary file 1 [file ijms-23-03390-s001.zip › FigS.pdf]

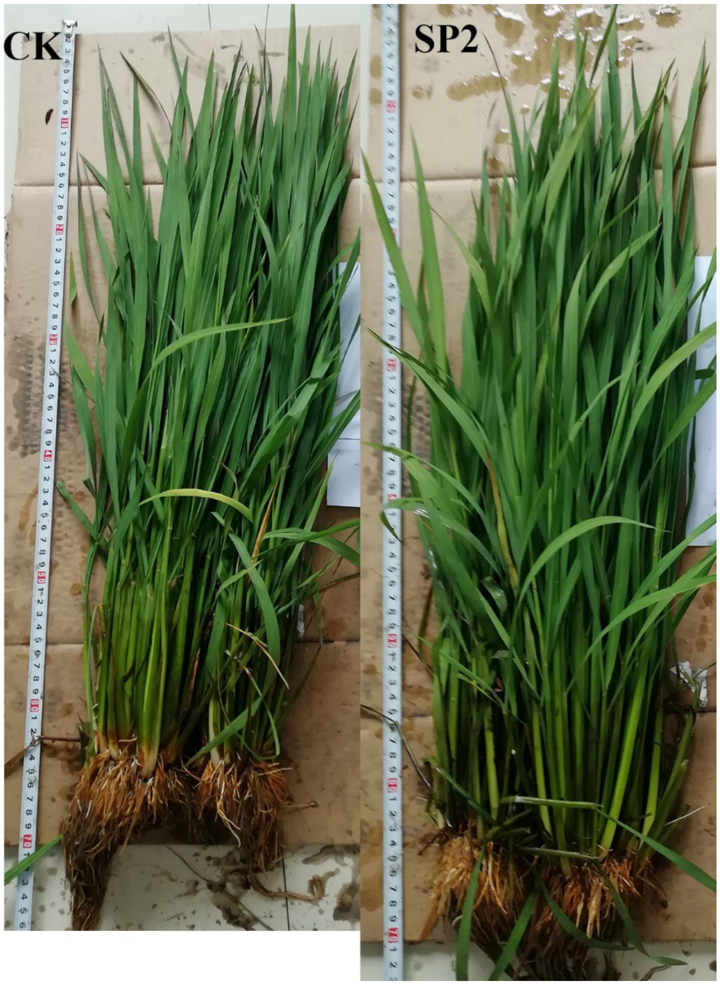

Figure S1. Photographs of progeny rice plants in TS.

A

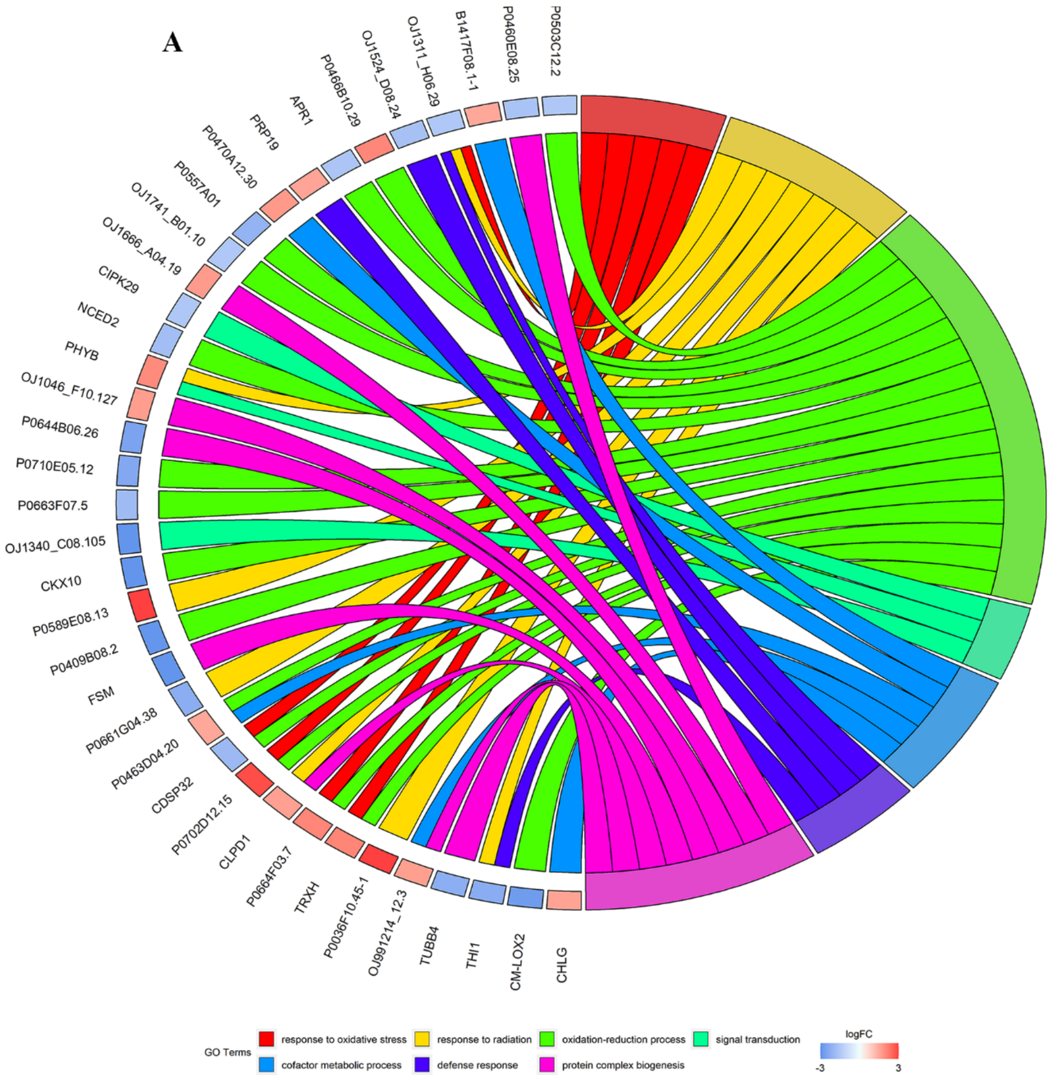
P0503C12.2
P0460E08.25
B1417F08.1-1
OJ1311_H06.29
OJ1524_D08.24
P0466B10.29
APR1
PRP19
P0470A12.30
P0557A01
OJ1741_B01.10
OJ1666_A04.19
CIPK29
NCED2
PHYB
OJ1046_F10.127
P0644B06.26
P0710E05.12
P0663F07.5
OJ1340_C08.105
CKX10
P0589E08.13
P0409B08.2
FSM
P0661G04.38
P0463D04.20
CDSP32
P0702D12.15
CLPD1
P0664F03.7
TRXH
P0036F10.45-1
OJ991214_12.3
TUBB4
THI1
CM-LOX2
CHLG
GO Terms
response to oxidative stress
response to radiation
oxidation-reduction process
signal transduction
cofactor metabolic process
defense response
protein complex biogenesis
logFC
-3
3

B

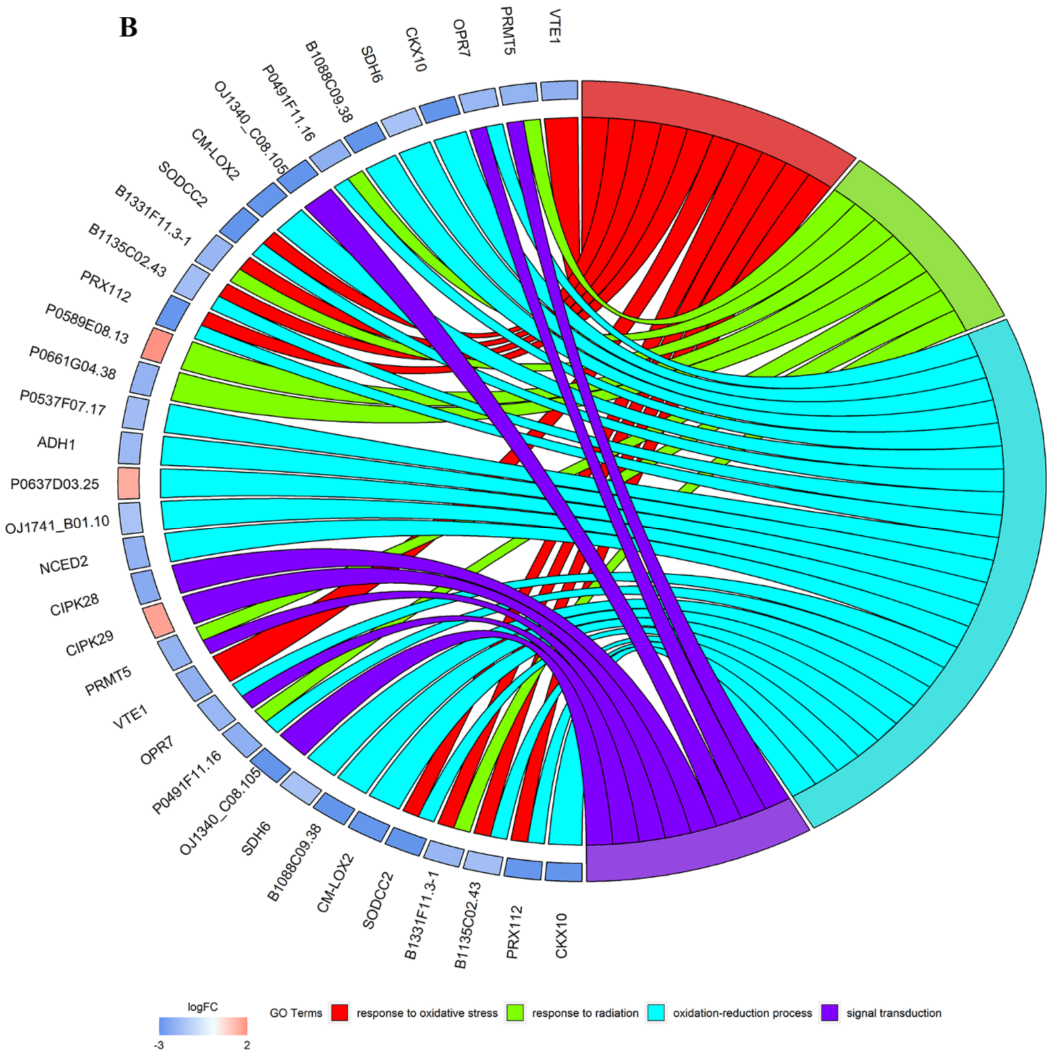

Figure S2. Chord diagram analyzes the interactions between BPs. A represents the different metabolites at the TLS; B represents the different metabolites at the TS; TLS and TS stand for three-leaf stage and tillering stage, respectively.

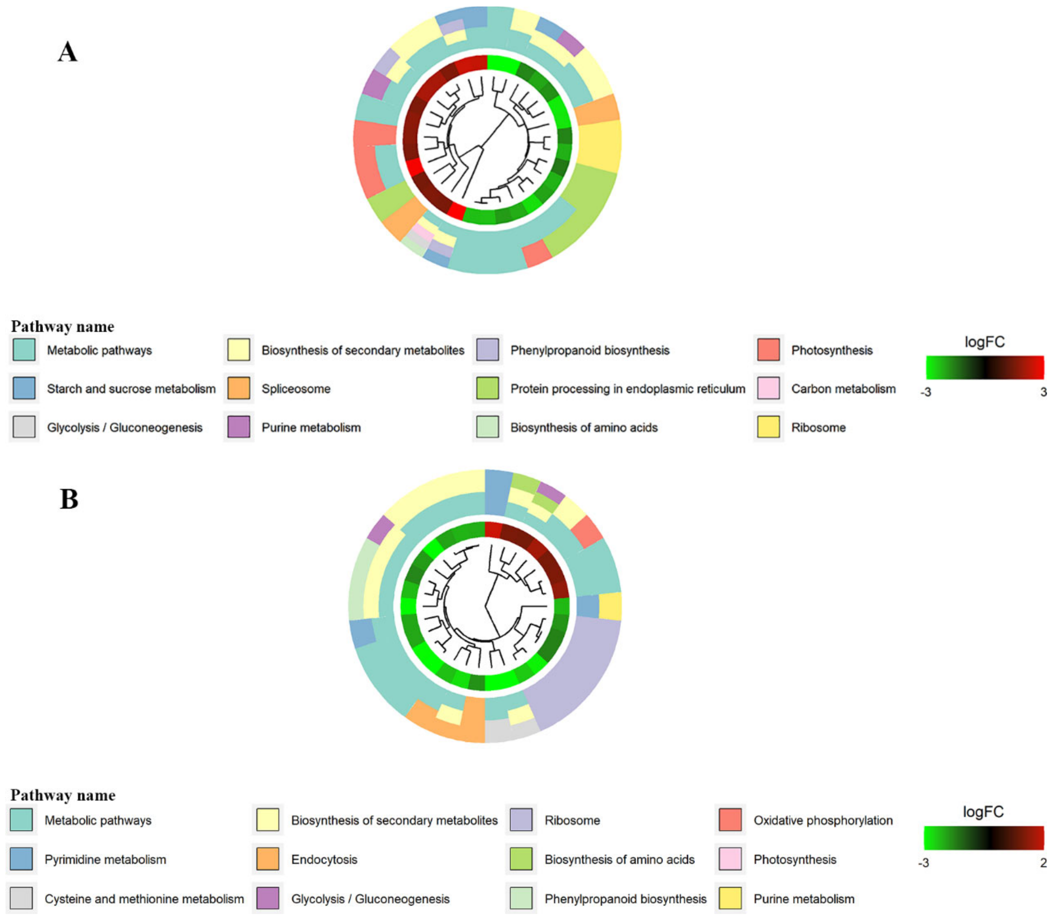

Figure S3. KEGG pathway enrichment analysis of proteins with differential abundance; TOP 12 of pathway enrichment; (A) three-leaf stage; (B) tillering stage.

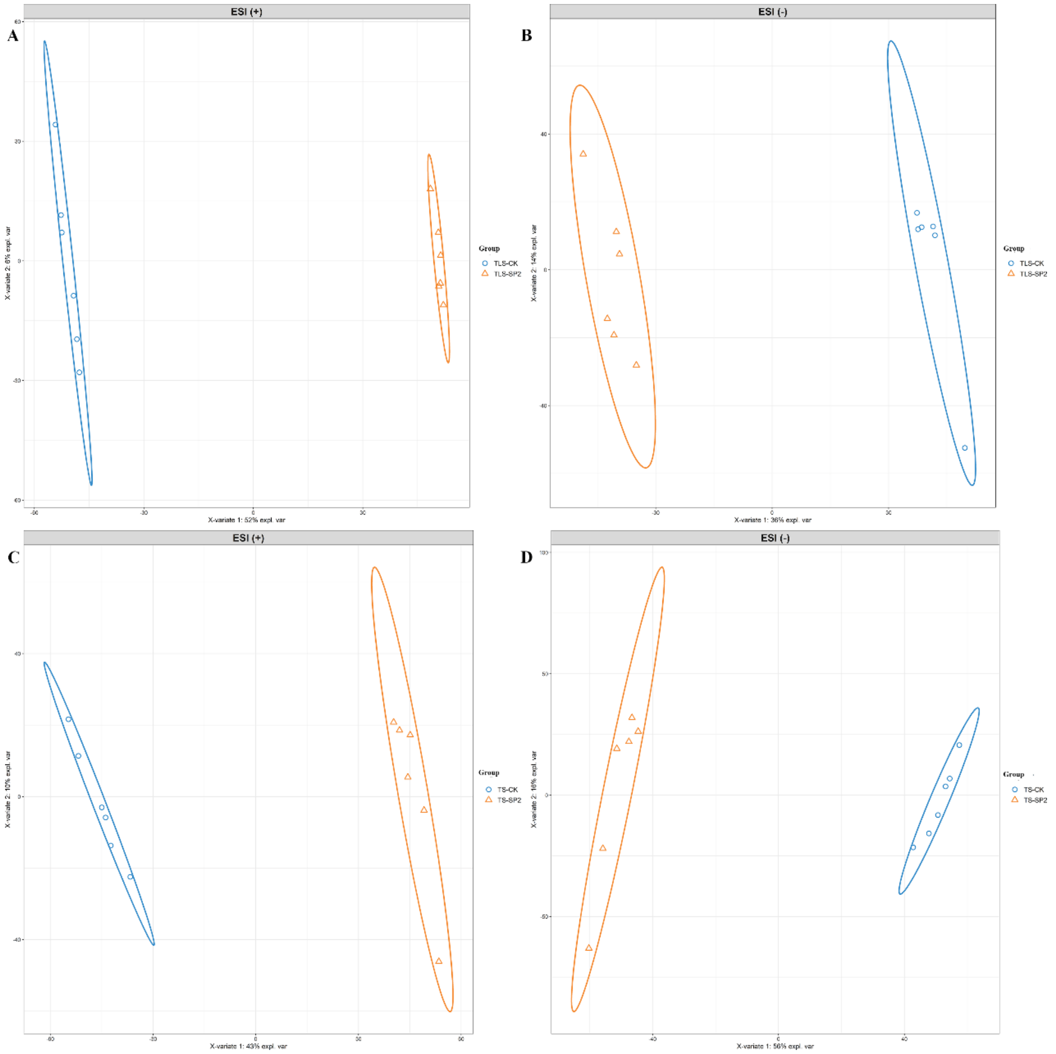

Figure S4. Partial least squares discriminant analysis (PLS-DA) score map of different rice sample groups based on UHPLC-QTOF/MS data (six biological replicates). (A) Positive ion mode of TLS, (B) Negative ion mode of TLS. (C) Positive ion mode of TS, (B) Negative ion mode of TS; TLS and TS represent three leaf period and tillering stage.CK represent control group. SP2 represent the offspring of rice after spaceflight.

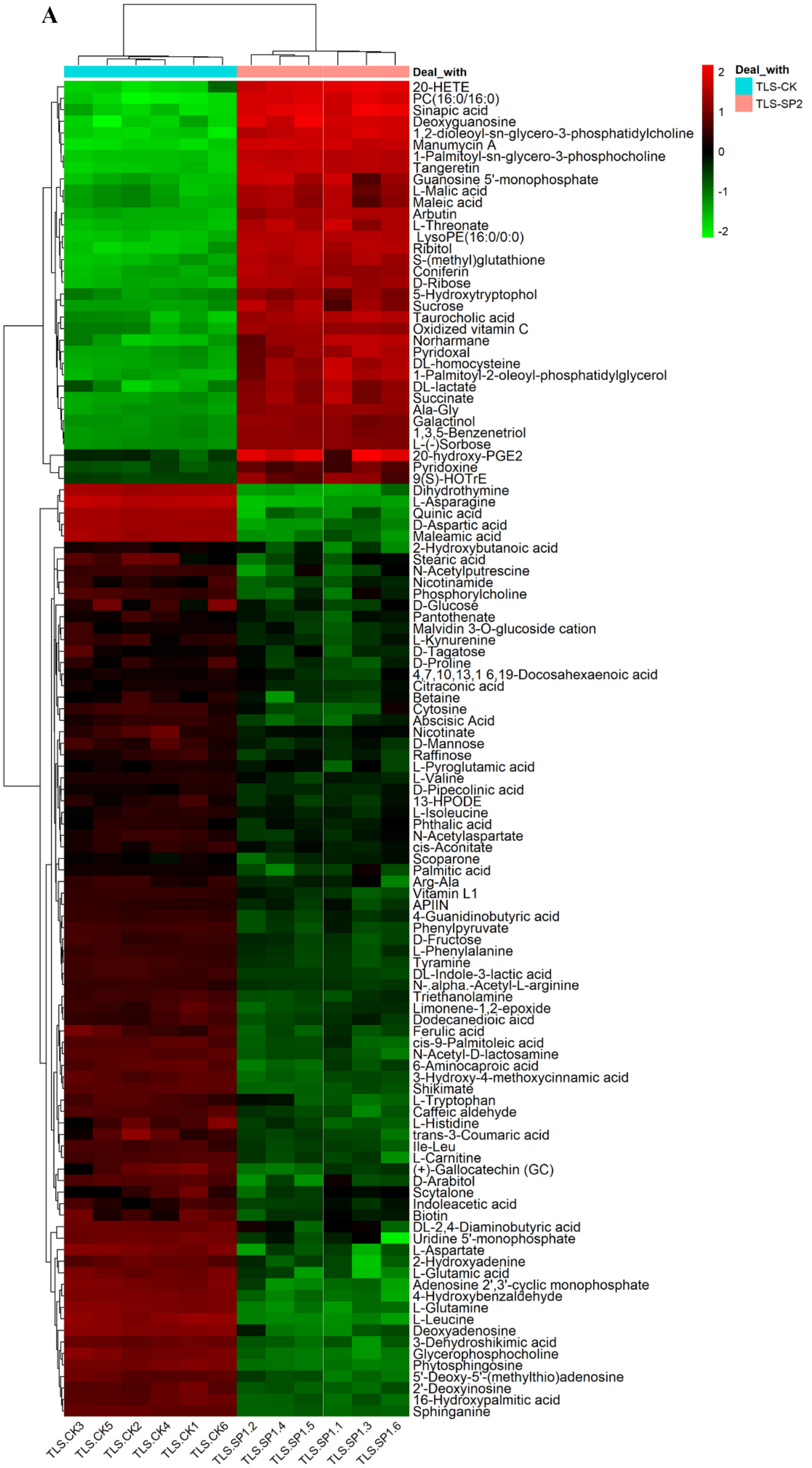
A
Deal_with
TLS-CK
TLS-SP2
2
1
0
-1
-2
20-HETE
PC(16:0/16:0)
Sinapic acid
Deoxyguanosine
1,2-dioleoyl-sn-glycero-3-phosphatidylcholine
Manumycin A
1-Palmitoyl-sn-glycero-3-phosphocholine
Tangeretin
Guanosine 5'-monophosphate
L-Malic acid
Maleic acid
Arbutin
L-Threonate
LysoPE(16:0/0:0)
Ribitol
S-(methyl)glutathione
Coniferin
D-Ribose
5-Hydroxytryptophol
Sucrose
Taurocholic acid
Oxidized vitamin C
Norharmane
Pyridoxal
DL-homocysteine
1-Palmitoyl-2-oleoyl-phosphatidylglycerol
DL-lactate
Succinate
Ala-Gly
Galactinol
1,3,5-Benzenetriol
L-(-)Sorbose
20-hydroxy-PGE2
Pyridoxine
9(S)-HOTrE
Dihydrothymine
L-Asparagine
Quinic acid
D-Aspartic acid
Maleamic acid
2-Hydroxybutanoic acid
Stearic acid
N-Acetylputrescine
Nicotinamide
Phosphorylcholine
D-Glucose
Pantothenate
Malvidin 3-O-glucoside cation
L-Kynurenine
D-Tagatose
D-Proline
4,7,10,13,1 6,19-Docosahexaenoic acid
Citraconic acid
Betaine
Cytosine
Abscisic Acid
Nicotinate
D-Mannose
Raffinose
L-Pyroglutamic acid
L-Valine
D-Pipecolinic acid
13-HPODE
L-Isoleucine
Phthalic acid
N-Acetylaspartate
cis-Aconitate
Scoparone
Palmitic acid
Arg-Ala
Vitamin L1
APIIN
4-Guanidinobutyric acid
Phenylpyruvate
D-Fructose
L-Phenylalanine
Tyramine
DL-Indole-3-lactic acid
N-.alpha.-Acetyl-L-arginine
Triethanolamine
Limonene-1,2-epoxide
Dodecanedioic aicd
Ferulic acid
cis-9-Palmitoleic acid
N-Acetyl-D-lactosamine
6-Aminocaproic acid
3-Hydroxy-4-methoxycinnamic acid
Shikimate
L-Tryptophan
Caffeic aldehyde
L-Histidine
trans-3-Coumaric acid
Ile-Leu
L-Carnitine
(+)-Gallocatechin (GC)
D-Arabitol
Scytalone
Indoleacetic acid
Biotin
DL-2,4-Diaminobutyric acid
Uridine 5'-monophosphate
L-Aspartate
2-Hydroxyadenine
L-Glutamic acid
Adenosine 2',3'-cyclic monophosphate
4-Hydroxybenzaldehyde
L-Glutamine
L-Leucine
Deoxyadenosine
3-Dehydroshikimic acid
Glycerophosphocholine
Phytosphingosine
5'-Deoxy-5'-(methylthio)adenosine
2'-Deoxyinosine
16-Hydroxypalmitic acid
Sphinganine
TLS.CK3
TLS.CK5
TLS.CK2
TLS.CK4
TLS.CK1
TLS.CK6
TLS.SP1.2
TLS.SP1.4
TLS.SP1.5
TLS.SP1.1
TLS.SP1.3
TLS.SP1.6

B

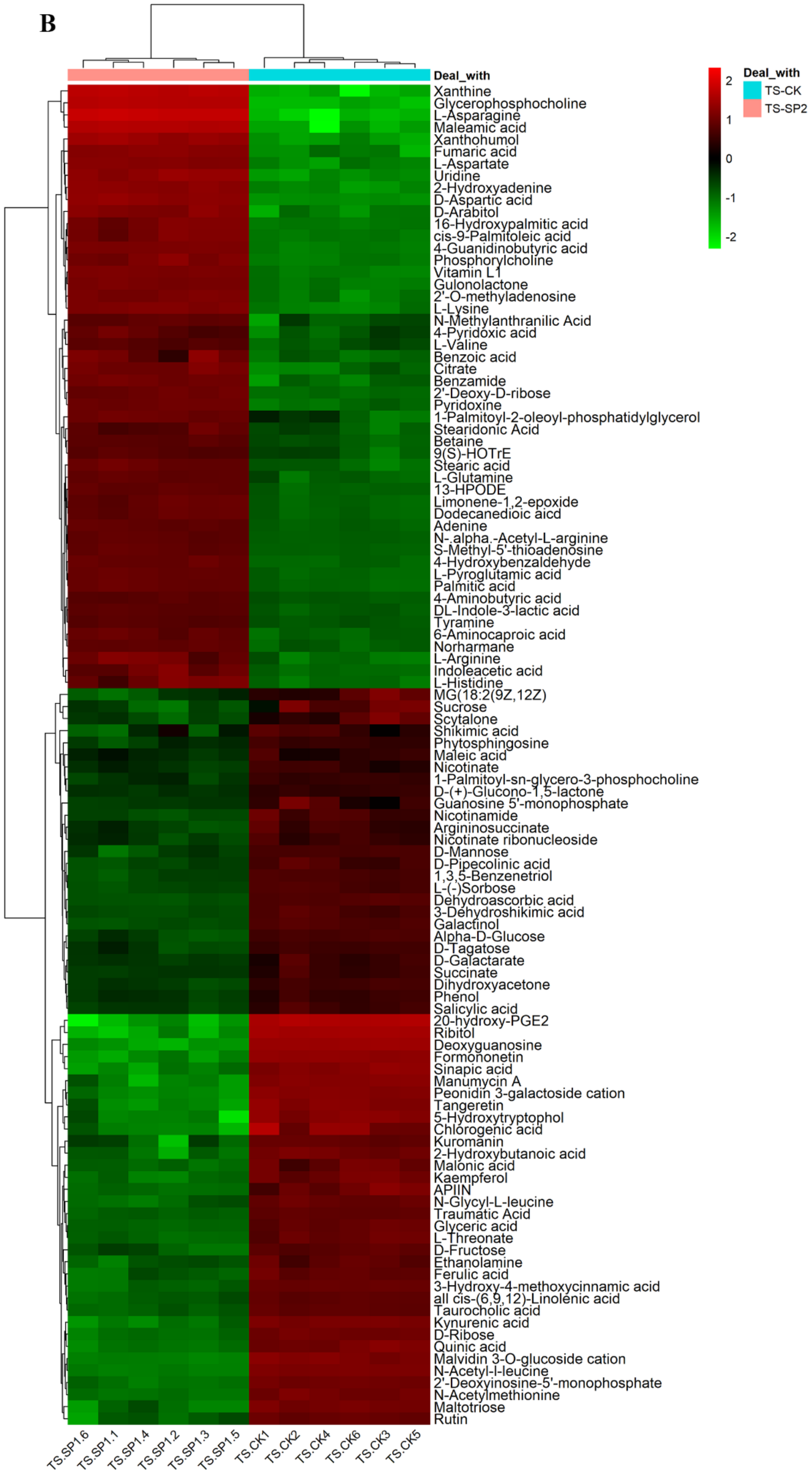

Figure S5. Results of hierarchical cluster analysis of changed metabolites pools. Hierarchical trees were drawn based on the detected changed metabolites in leaves of rice, (A) TLS, (B) TS. Columns correspond to the

repetition between different treatments, while rows represent different metabolites. Red and green colors indicate increased and decreased metabolite concentrations, respectively.

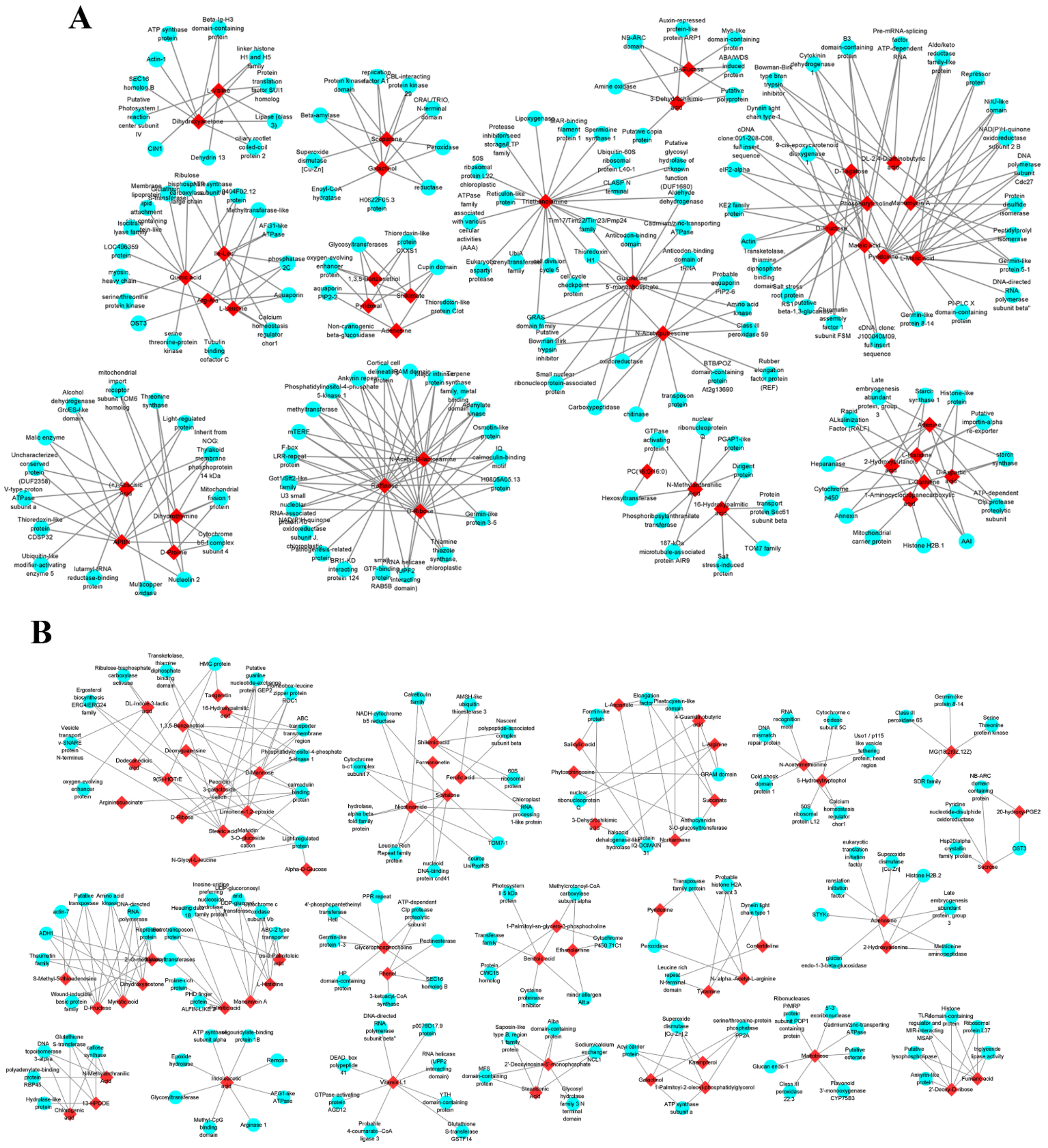

Figure S6. Analysis of the relationship between proteomics and metabolomics. The psych package of R software is used for analysis, and cytoscap was used for visualization. Red represents protein and blue represents metabolites.

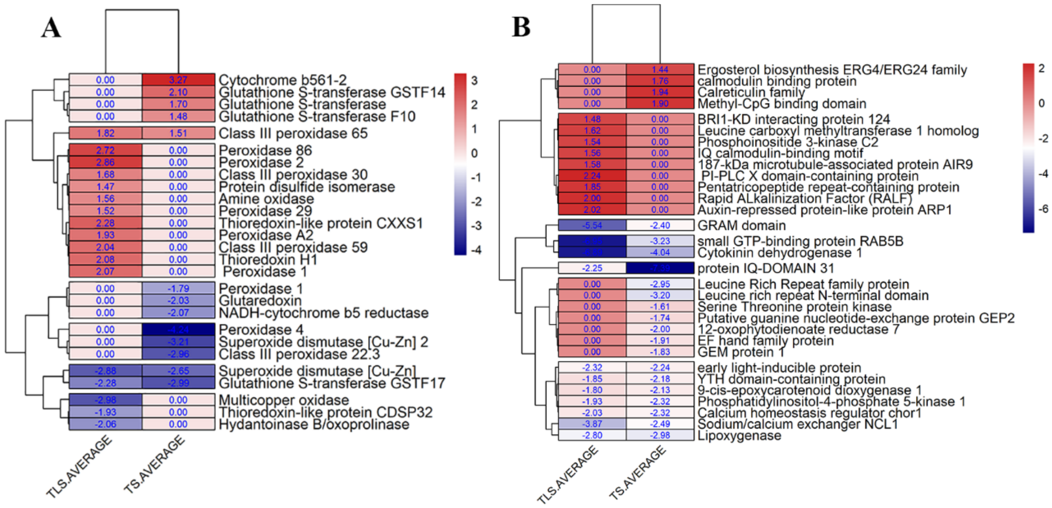

Figure S7. DAPs involved in redox reactions and signal transduction processes. A represents the DAPs involved in the redox reaction. B represents the DAPs involved in the signal transduction process. The number represents $\log_{1.2}^{(FC)}$, a positive number represents an up-regulated protein, a negative number represents a down-regulated protein, and 0.00 represents a protein that is not significantly different.

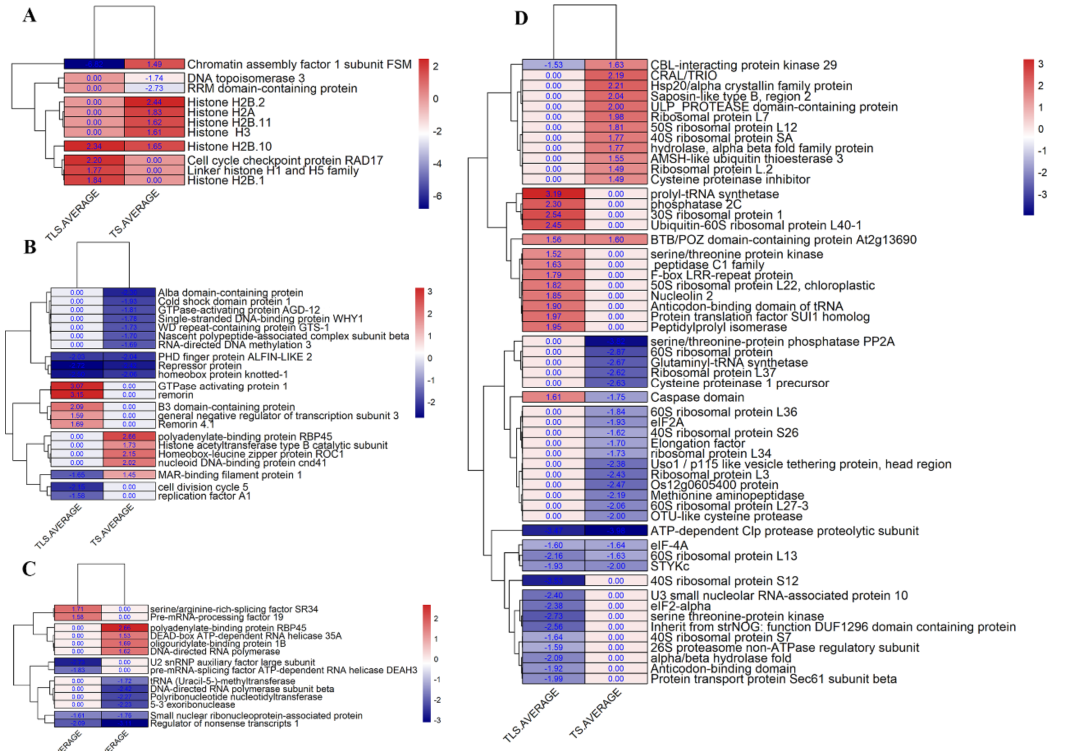

Figure S8. Genetic material metabolism and protein reorganization are the key processes of offspring rice coping with spaceflight. A represents the DAPs involved in the DNA. B represents the change of transcription factors. C represents DAPs involved in RNA processing. D represents DAPs in protein synthesis, processing and degradation. The number represents $\log_{1.2}^{(FC)}$, a positive number represents an up-regulated protein, a negative number represents a down-regulated protein, and 0.00 represents a protein that is not significantly different.

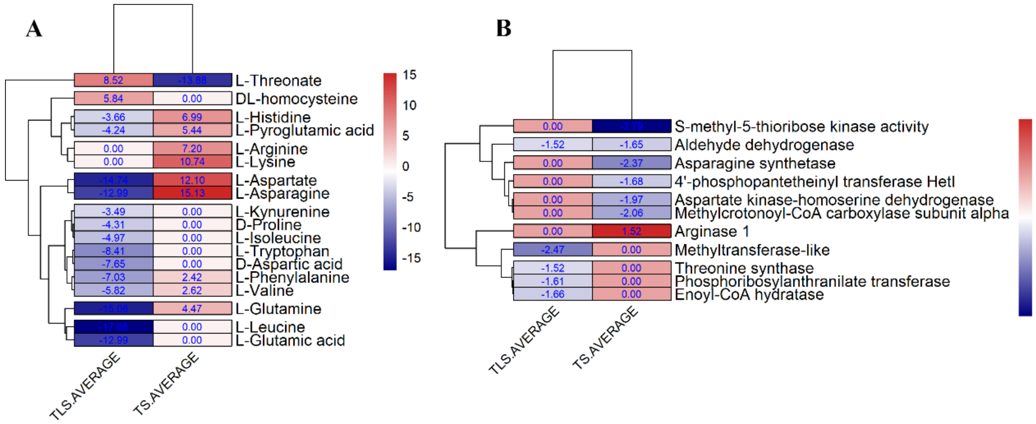

Figure S9. Spaceflight changed the amino acid metabolism profile of offspring rice. A represents the DAPs involved in amino acid metabolism. B represents DAPs involved in amino acid metabolism. The number represents $\log_{1.2}{}^{(FC)}$, a positive number represents an up-regulated protein, a negative number represents a down-regulated protein, and 0.00 represents a protein that is not significantly different.

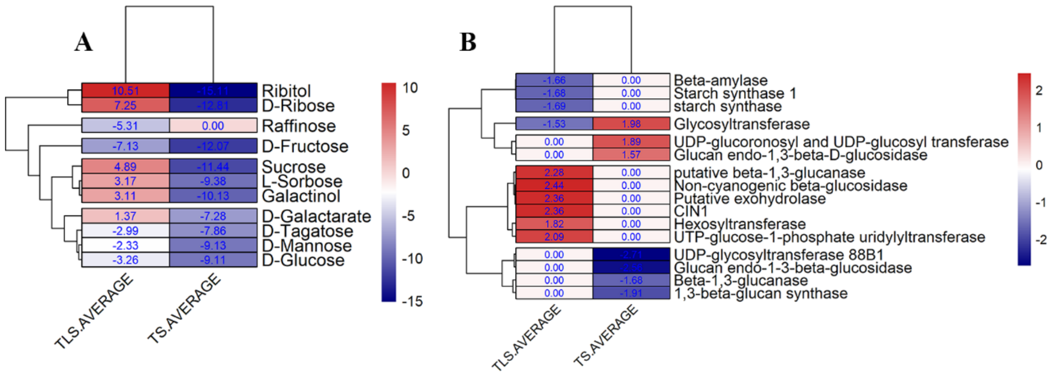

Figure S10. Spaceflight changed the carbohydrate metabolism profile of offspring rice. A represents the DAPs involved in carbohydrate metabolism. B represents DAPs involved in carbohydrate metabolism. The number represents $\log_{1.2}{}^{(FC)}$, a positive number represents an up-regulated protein, a negative number represents a down-regulated protein, and 0.00 represents a protein that is not significantly different.

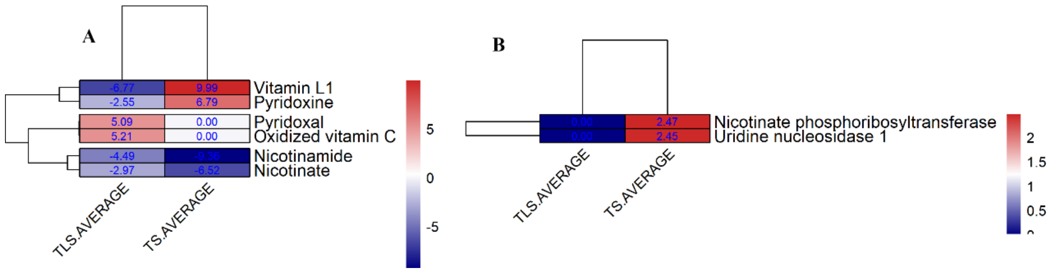

Figure S11. Spaceflight changed the vitamin and cofactor metabolism profile of offspring rice. A represents the DAPs involved in vitamin and cofactor metabolism. B represents DAPs involved in vitamin and cofactor metabolism. The number represents $\log_{1.2}{}^{(FC)}$, a positive number represents an up-regulated protein, a negative number represents a down-regulated protein, and 0.00 represents a protein that is not significantly different.
