# Supplementary material for: The Memory of Rice Response to Spaceflight Stress: From the Perspective of Metabolomics and Proteomics
Source: Int J Mol Sci. 2022 Mar 21;23(6):3390. doi: 10.3390/ijms23063390 (PMC8954569; doi:10.3390/ijms23063390)
Supplement: Supplementary file 1 [file ijms-23-03390-s001.zip › Table S8.pdf]

Table S8. Primers used in the qRT-PCR analysis.

name	Forward primer (5'–3')	Reverse primer (5'–3')
Superoxide dismutase [Cu-Zn]	GGTGTAGCTGAGGCAACCAT	AACAACACCGCATGCAAGTC
glutathione transferase	CCTACTGGCTGGAGGTGCTA	CGCGACAGCAACATTATCCC
Sodium/calcium exchanger NCL1	CAGCTATGCAGCTCGGATCA	TAGGCAAGCACAAGCGAGAA
Calcium homeostasis regulator chor1	TTCAGCGAGTGTATGAGCCG	GGAACTCCTTGGAGTGTGGG
GRAM domain	CGCCTGTGACCAATACTCA	TACCGCGTGATCGTCATCAG
Class III peroxidase 65	CTGATTCCGTTGGAGGCTAGG	ATCTCCATCTCGAAGCGGTG
